# An Exploration of Nanobiotechnology Bridging Patho-Therapeutics with Regenerative and Clinical Perspectives in Periodontitis

**DOI:** 10.3390/jfb17010045

**Published:** 2026-01-15

**Authors:** Baozhu Zhang, Muhammad Umar Javed, Yinghe Zhang, Bing Guo

**Affiliations:** 1 Department of Radiation Oncology, The People’s Hospital of Baoan, The Second Affiliated Hospital of Shenzhen University, Shenzhen 518101, China; 2 School of Science, Harbin Institute of Technology, Shenzhen 518055, China; muhammadumarpusc@gmail.com; 3 Shenzhen Key Laboratory of Advanced Functional Carbon Materials Research and Comprehensive Application, Harbin Institute of Technology, Shenzhen 518055, China; caroline.w.2006@gmail.com

**Keywords:** periodontitis, nanomaterials, immunotherapy, ROS scavenging, gas therapy

## Abstract

Periodontal disease represents a major global concern characterized by chronic biofilm-driven inflammation, excessive oxidative stress, progressive tissue destruction, and impaired regenerative capacity. Beyond conventional antimicrobial approaches, recent progress has shifted toward host-directed and regenerative therapeutic strategies aimed at restoring both oral function and tissue homeostasis. This review consolidates current developments in nanobiotechnology-based materials that modulate immune responses, scavenge reactive oxygen species, and promote angiogenesis and osteogenesis, thereby facilitating the effective regeneration of dental and periodontal tissues. Emphasis is placed on bioresponsive hydrogels, bioactive scaffolds, and gas-releasing platforms that integrate therapeutic regulation with tissue repair. The discussion further highlights key advances in polymeric and inorganic biomaterials designed to balance antibacterial action with cellular compatibility and regenerative potential. By linking pathophysiological mechanisms with material-guided healing processes, this review provides a comprehensive perspective on emerging nanobiotechnological solutions that bridge patho-therapeutics with regenerative and clinical dentistry.

## 1. Introduction

Oral health is essential to overall well-being, influencing systemic health, nutrition, quality of life, and social interaction. However, oral diseases, ranging from dental caries and periodontitis to oral mucosal lesions and fungal infections, remain among the most prevalent and troublesome worldwide [1,2]. Bacterial and non-bacterial infections present significant clinical challenges due to their multifactorial origins, complex pathological mechanisms, and growing resistance to conventional therapies [3]. Despite major advances in preventive dentistry and public health awareness, the global incidence of oral infections continues to rise, driven by modern lifestyle factors, an aging population, and the increasing prevalence of chronic diseases [4]. Periodontitis represents one of the most prevalent chronic inflammatory diseases causing tooth loss in adults [5]. It is characterized by a dysregulated host–microbial interaction that results in progressive destruction of the periodontal ligament, alveolar bone resorption, and ultimately compromised oral function. Unlike acute oral infections, periodontitis develops within a complex and dynamic ecological niche shaped by multispecies biofilms, host immune responses, salivary components, and mechanical forces [6,7].

As the biofilm matures, a shift from symbiotic to dysbiotic microbial communities occurs, characterized by the enrichment of anaerobic and proteolytic species. *Porphyromonas gingivalis* (*P. gingivalis*), a keystone pathogen in periodontitis, causes immune subversion and tissue degradation [8,9]. Moreover, fungal species such as *Candida albicans* (*C. Albicans*), particularly in immunocompromised or dysbiotic environments, can enhance biofilm complexity and inflammation through morphological plasticity and immune evasion mechanisms [9,10]. These microbial agents often coexist in biofilm communities, structured, resilient microbial aggregates that confer protection from host defenses and antibiotics, making treatment considerably more difficult. A comprehensive understanding of their pathological mechanisms is essential to address these infections effectively. For instance, *P. gingivalis* expresses gingipains that cleave host immune proteins and manipulate signaling pathways to sustain inflammation and evade clearance [11]. *S. mutans* produces extracellular polysaccharides that enhance adhesion and biofilm formation, driving demineralization of enamel [12]. *C. albicans* transitions between yeast and hyphal forms, enabling tissue invasion and immune evasion [13]. Such pathogenic versatility underscores the limitations of conventional therapies like antibiotics and antifungals, which often fail to eradicate infections, especially within biofilms, fully, and may contribute to the emergence of resistant strains.

Clinically, periodontitis is managed primarily through mechanical debridement aimed at disrupting subgingival biofilms and removing necrotic tissue. Adjunctive antibiotic therapy is reserved for selected cases and is increasingly constrained by limited penetration into biofilm matrices, enzymatic inactivation, and rising antimicrobial resistance [14,15]. Prolonged or inappropriate courses foster the emergence of resistant bacteria, disturb protective oral flora, and predispose to secondary infections by multi-resistant organisms or yeasts [16]. The efficacy of antibiotics is further compromised by the presence of necrotic tissue, pus, and β-lactamase activity within lesions, which inactivate many agents if surgical management is delayed [17]. Rising resistance rates, such as penicillin resistance in oral anaerobes and macrolide resistance in *Viridans streptococci,* alongside cross-reactivity between drug classes, significantly restrict therapeutic options. Even short antibiotic exposure carries risks, including candidiasis, gastrointestinal upset, pseudomembranous colitis, and rare but life-threatening anaphylaxis. Each cycle also contributes to long-term colonization by resistant organisms, complicating both individual patient care and broader public health [18,19,20].

In light of these challenges, recent years have witnessed the emergence of advanced therapeutic strategies that harness the principles of materials science, nanotechnology, and precision medicine [21]. Among these, photothermal therapy (PTT) [22], photodynamic therapy (PDT) [23], chemodynamic therapy (CDT) [24], and sonodynamic therapy (SDT) [25] offer promising non-antibiotic approaches to eradicate bacterial infections through physical or chemical disruption mechanisms [26]. These methods exploit exogenous or endogenous stimuli, such as light, ultrasound (US), or hydrogen peroxide (H_2_O_2_), to activate nanoparticles that generate heat or reactive oxygen species (ROS), leading to bacterial cell death and biofilm breakdown [27,28]. While these pathogen-targeted strategies aim to eradicate microbial sources of infection, long-term recovery in oral diseases also requires restoring tissue homeostasis and immune balance. Therefore, host-directed and regenerative therapies have emerged as complementary approaches that modulate inflammatory responses, redox state, and tissue repair processes [29,30]. In such cases, therapeutic strategies focus on antioxidant delivery, ROS-scavenging nanomaterials, and drug delivery systems tailored to the affected tissue microenvironment [31,32]. Notably, gas therapy using nitric oxide (NO) and hydrogen sulfides (H_2_S) donors has emerged as a promising adjunct for regulating inflammation, promoting healing, and restoring redox balance [33,34]. Recent progress in biomaterials engineering has significantly expanded the scope and specificity of these treatments [35,36]. Natural and synthetic polymeric hydrogels, refs. [37,38] stimuli-responsive nanocarriers, and bioactive inorganic nanomaterials offer tailored solutions for targeted delivery, controlled release, and site-specific activation [39]. These smart biomaterials are designed to adapt to the unique physicochemical characteristics of the oral microenvironment, whether acidic pH, high glutathione (GSH) levels, or abundant H_2_O_2_, ensuring localized and effective therapy with minimal systemic toxicity [40,41]. Dental tissue loss resulting from infectious diseases, trauma, or chronic inflammation also poses a significant therapeutic challenge [42]. Conventional treatments, including prosthetics and surgical interventions, primarily aim at functional replacement without restoring native tissue architecture or biological function [43]. In recent years, regenerative strategies have gained considerable attention, aiming to restore the vitality and structural integrity of teeth and their supporting tissues. Central to these approaches is the use of scaffolds, signaling molecules, and stem or progenitor cells that collectively facilitate tissue repair [44,45]. Moreover, addressing the underlying oxidative stress and inflammatory responses has proven crucial for improving the regenerative microenvironment [46]. For nanomaterials and advanced biomaterials introduced into the periodontal environment, pellicle formation represents a critical determinant of in situ behavior [47,48,49]. Protein adsorption can modify surface charge, hydrophobicity, and ligand accessibility, thereby influencing cellular uptake, antimicrobial efficacy, and drug release kinetics. In some cases, uncontrolled bio-adsorption may attenuate therapeutic activity or promote unintended microbial adhesion [50,51]. Conversely, rationally engineered materials that account for pellicle interactions may leverage selective protein binding to enhance retention at diseased sites or improve biocompatibility. Recognizing and integrating pellicle-mediated effects is thus essential for translating nanotherapeutics from simplified in vitro systems to clinically relevant periodontal conditions [52]. With growing insights into cell-material interactions and immune modulation, research is now moving toward the development of integrated platforms that can simultaneously support tissue regeneration, control inflammation, and enhance long-term oral health.

Furthermore, with the integration of artificial intelligence (AI) into dental research and practice, diagnostic accuracy, treatment personalization, and clinical efficiency have reached unprecedented levels [53]. AI-driven models now assist in the early detection of lesions through image recognition, predictive modeling of disease progression, and optimization of treatment protocols [54]. In dental materials research, machine learning (ML) algorithms assist in the discovery and evaluation of novel nanoplatforms by predicting biological interactions and therapeutic outcomes, significantly reducing the time and cost of experimental validation [55,56].

Given the rapid pace of innovation, numerous reviews have been published on the therapeutic strategies or material platforms. However, there is a notable lack of comprehensive analyses that explore the pathological continuum of oral infections, from microbial colonization to tissue inflammation, and that connect novel nanomaterial-based therapies to clinical outcomes.

This review seeks to fill that gap by offering a consolidated perspective on the state-of-the-art host-directed and regenerative therapeutic strategies for periodontitis. It outlines the intricate pathogenesis of key oral pathogens, discusses recent material and therapeutic innovations, and presents a framework for multimodal treatment approaches that address the multifactorial nature of oral disease. This review aims to guide future investigations, inspire translational efforts, and ultimately contribute to more effective, personalized, and sustainable oral healthcare solutions by critically evaluating recent developments in preclinical and clinical research.

## 2. Pathological Mechanisms

**Figure 1:**
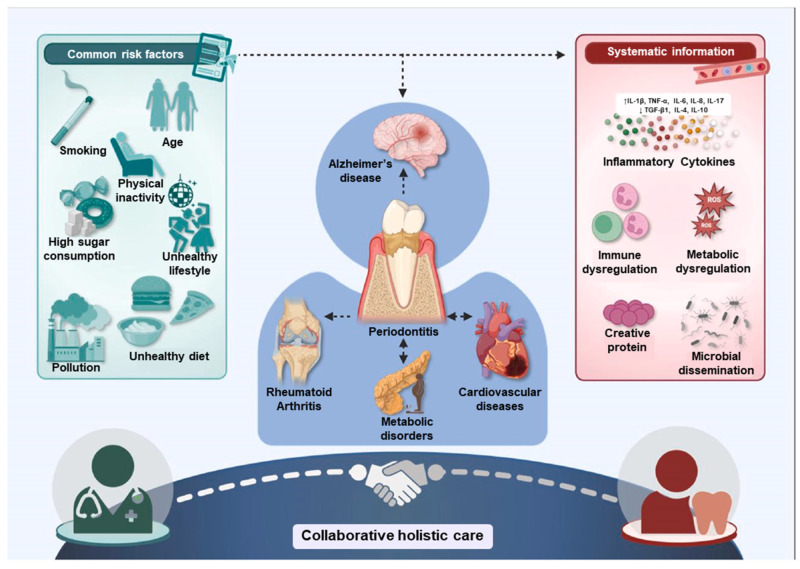
Molecular and immunological links between oral and systemic health [62]. Reprinted from Ref. [62].

Oral health is now widely acknowledged as an essential aspect of overall well-being, significantly influencing various systemic functions [57,58]. Among oral diseases, periodontitis, a chronic inflammatory disorder affecting the gums and supporting structures of the teeth, has been increasingly linked to broader health consequences [59]. Managing periodontitis not only preserves oral integrity but also contributes to alleviating the burden of systemic chronic diseases, leading to better clinical outcomes and reduced healthcare expenditures [60]. Routine oral health evaluations, especially for individuals with underlying conditions such as cardiovascular disease or diabetes, should be incorporated into standard medical care protocols [61]. Disruptions in the balance of the oral microbiota often allow pathogenic species to proliferate, initiating a cascade of dental disorders [62]. The pathogenic shift begins with the accumulation of dental plaque, a microbial biofilm that adheres to tooth surfaces. When not effectively removed through oral hygiene, this plaque calcifies into calculus, creating a hospitable environment for bacterial colonization [63]. These bacteria elicit immune responses characterized by the release of pro-inflammatory mediators, including interleukin-1 (IL-1), tumor necrosis factor-alpha (TNF-α), and matrix metalloproteinases (MMPs), which contribute to the degradation of connective tissue and alveolar bone. Periodontitis extends beyond a localized oral infection, exerting systemic effects through sustained inflammation [64]. This chronic inflammatory state plays a contributory role in the development of systemic conditions such as cardiovascular disease, diabetes, rheumatoid arthritis, and neurodegenerative disorders like Alzheimer’s disease [65]. Elevated systemic markers, such as C-reactive protein (CRP) and interleukin-6 (IL-6), are frequently observed in both periodontitis and these comorbidities, indicating a convergence of pathological pathways [66]. Considering the interconnected nature of oral and systemic health, timely identification, prevention, and management of periodontitis are imperative. Consistent dental care, alongside diligent oral hygiene, can markedly reduce the risk of periodontitis and its broader health implications [67,68]. This underscores the value of integrated healthcare approaches, encouraging collaboration between dental and medical practitioners to comprehensively address the dual dimensions of patient health. (Figure 1) [62].

The oral cavity represents a dynamic microbial ecosystem, hosting over 770 bacterial species along with various viruses and fungi, each contributing to either oral homeostasis or disease [69]. Understanding the mechanisms by which these microorganisms become pathogenic, and the individual and environmental factors influencing susceptibility, is essential for developing targeted interventions [70]. Common oral residents such as *S. mutans*, *Porphyromonas gingivalis* (*P. gingivalis*), *Treponema denticola* (*T. denticola*), and *C. albicans* are frequently implicated in dental pathologies [71]. The composition of the oral microbiota varies among individuals due to influences including diet, hygiene, genetic makeup, and environmental exposure. Perturbations such as smoking, antibiotic use, or systemic infections can disrupt microbial balance, leading to dysbiosis and the emergence of pathogenic phenotypes. When commensal microorganisms transition into virulent forms, they evade immune defenses and initiate disease processes [72]. Plaque biofilm accumulation on tooth surfaces serves as the primary site for microbial colonization. If not regularly removed, it mineralizes into calculus, promoting bacterial persistence [73,74,75]. These pathogens elicit host immune responses, triggering pro-inflammatory mediators such as IL-1, TNF-α, and MMPs, which contribute to connective tissue breakdown and alveolar bone resorption. Acquired pellicle, which is a thin layer of adsorbed salivary proteins and glycoproteins, also plays a pivotal role in periodontal pathogenesis by acting as the initial substrate for microbial adhesion and biofilm maturation [76,77]. The composition and conformation of pellicle proteins influence bacterial selectivity, biofilm architecture, and the subsequent activation of host immune responses. In periodontitis, alterations in pellicle composition, driven by inflammation, enzymatic degradation, and microbial metabolism, can exacerbate dysbiosis and perpetuate tissue destruction [78,79]. Periodontal intervention must consider the pellicle as a dynamic biological interface rather than a passive surface coating. Oral pathogens exhibit diverse morphologies suited to their environmental niches and are key contributors to conditions like dental caries and periodontal disease. Among the most prominent are *P. gingivalis*, *Aggregatibacter actinomycetemcomitans* (*A. actinomycetemcomitans*), *Tannerella forsythia* (*T. forsythia*), and *T. denticola*, particularly in the context of periodontitis [80]. Homeostatic balance in the oral microbiome is sustained through mutualism between the host and microbes. However, a shift toward parasitism underlies disease onset, particularly in caries and inflammatory periodontal conditions [81,82].

The oral microbiota is highly individual, shaped by age, genetics, health status, lifestyle, and geographic factors, rendering it as unique as a fingerprint. This variability influences disease risk and necessitates personalized approaches to prevention and care [83]. Current data indicate that dominant pathogens by prevalence include herpes viruses (60%), *C. albicans* (22%), *S. mutans* (6%), *P. gingivalis* (5%), and others [81]. *C. albicans* remains the primary fungal agent of oral candidiasis, though related species like *Candida glabrata* (*C. glabrata*) and *Candida dubliniensis* (*C. dubliniensis*) are also implicated. Pathogenesis involves fungal adhesins such as ALS3 and HWP1 binding to epithelial receptors (e.g., E-cadherin, EphA2), enabling biofilm formation and resistance to antifungals. Co-aggregation with bacteria like *S. gordonii* further stabilizes biofilms via protein–protein interactions (ALS3-SspB). The hyphal transition enhances tissue invasion, aided by thigmotropism and the secretion of degradative enzymes (SAPs, phospholipases). Candidalysin, a hypha-specific toxin, exacerbates host cell damage, while internalization into host cells is mediated by ALS3 and Ssa1 through degradation of intercellular junctions [84]. The oral microbiota’s influence extends beyond local pathology. In rheumatoid arthritis (RA), microbial dysbiosis has been linked to disease initiation and progression. *P. gingivalis* contributes via its peptidylarginine deiminase (PPAD), which induces citrullination of host proteins, a process central to ACPA production in RA. Neutrophil extracellular traps (NETs) generated during infection further amplify this effect via PAD4. Dysbiotic profiles characterized by elevated *Prevotella*, *Veillonella*, and *Lactobacillus salivarius* (*L. salivarius*), along with reduced *Haemophilus* spp., promote Th17-mediated inflammation. *A. actinomycetemcomitans* has also been implicated, with leukotoxin-A driving neutrophil hypercitrullination. Molecular mimicry, such as that between *P. gingivalis* α-enolase and its human counterpart, may further perpetuate autoimmune activation [84].

This interrelation of oral and systemic health underscores the importance of microbial equilibrium in the oral cavity. Microbial translocation between the oral and gut microbiomes supports a bidirectional model, wherein disturbances in one niche may provoke systemic consequences [85]. As evidence accumulates, maintaining oral microbial balance is increasingly recognized as integral to whole-body health. Advancements in this field support a multidisciplinary approach, incorporating evidence-based strategies such as the use of dental probiotics (*S. salivarius*, *Lactobacillus* spp.), xylitol-containing formulations, fluoride-based products, and essential oil mouth rinses [86,87]. Continued exploration of novel oral health products and cross-disciplinary methodologies will be key to enhancing our understanding of the oral microbiota and developing targeted, effective interventions.

## 3. Host-Directed and Regenerative Therapeutic Strategies

Bacterial infections and non-bacterial pathogens such as fungi, viruses, and parasites present significant health challenges. The growing prevalence of drug-resistant strains among these pathogens highlights the urgent need for innovative therapeutic strategies. One such strategy involves the use of ROS scavengers, which can neutralize the oxidative damage caused by ROS during infection. This can help restore the balance of the body’s immune response and improve overall health outcomes. Another innovative approach is the development of advanced drug delivery systems. These arrangements can enhance the efficacy of antifungal and antiviral agents by ensuring precise delivery to the site of infection while minimizing side effects. Additionally, combining ROS scavenging with gas therapies, such as nitric oxide or carbon monoxide, offers a multifaceted approach to combat infections. These gases can improve the local microenvironment and regulate the immune response, thereby enhancing the effectiveness of the treatment. These emerging strategies hold great potential for addressing the growing threat of non-bacterial infections in various clinical settings.

### 3.1. Immunotherapy

Immunotherapy is a promising approach in managing periodontitis and periapical pathologies, where dysregulated host immune responses play a central role in disease progression [88]. Instead of relying solely on antimicrobial elimination, immunotherapeutic strategies aim to restore the balance between pro-inflammatory and anti-inflammatory pathways, thereby preserving tissue integrity and promoting regeneration [89]. By modulating the activity of immune cells such as macrophages, T cells, and dendritic cells, and targeting key signaling cascades like NF-κB, it is possible to reduce excessive inflammation while supporting reparative processes [90]. Biomolecules, bioactive proteins, and engineered materials are being explored for their ability to suppress destructive immune activation, enhance regulatory mechanisms, and foster an environment conducive to healing. Such approaches may not only improve the outcomes of conventional treatments but also pave the way for vital pulp preservation, regeneration of periodontal structures, and long-term stability of dental tissues [91]. Ultimately, immunotherapy holds the potential to shift dental care from purely infection-eliminating strategies toward precision-based, host-directed interventions that integrate pathogen control with immune modulation for durable oral health.

He et al. [92] reported the development of a three-dimensionally printed bioactive glass ceramic scaffold doped with molybdenum ions (Mo-BGC), designed as an immunomodulatory platform for periodontal regeneration. The scaffold was fabricated with a precisely engineered hollow-pipe architecture, which enabled sustained and high-level release of bioactive ions from the Mo-BGC matrix, thereby supporting prolonged biological activity in vivo. Beyond structural design, incorporation of Mo ions endowed the scaffold with bioenergetic functionality, allowing modulation of mitochondrial metabolism and thereby directing macrophage phenotypic transitions.

**Figure 2:**
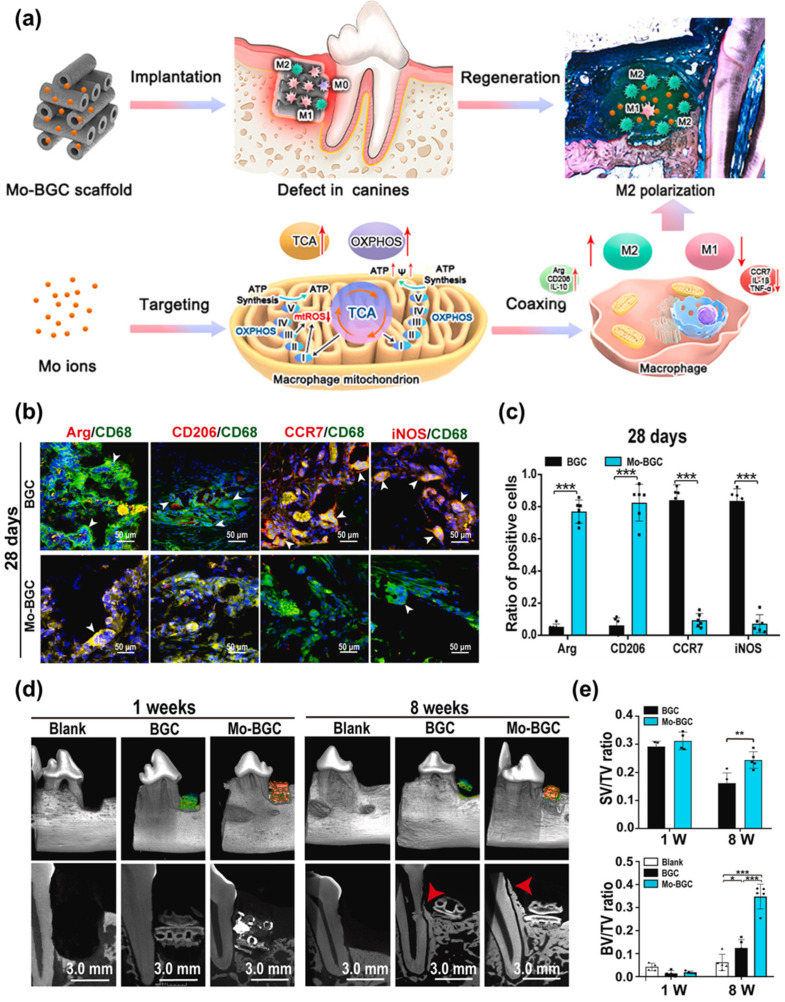
(**a**) Schematic illustration showing local macrophage polarization induced by implantation of a Mo-BGC scaffold within a periodontal bone defect. (**b**,**c**) Immunohistochemical and molecular analyses at 28 days post-implantation demonstrate enhanced expression of M2-associated markers alongside suppressed M1-associated markers in Mo-BGC scaffolds, indicating sustained immunoregulatory activity, *** *p* < 0.001 indicate significant differences between the indicated columns (two-way ANOVA). (**d**) Representative micro-CT reconstructions revealing newly formed alveolar bone within canine periodontal defects treated with undoped BGC and Mo-BGC scaffolds at 8 weeks after surgery. (**e**) Quantitative micro-CT evaluation of bone microarchitecture parameters, including structure volume fraction (SV/TV), bone volume fraction (BV/TV), trabecular thickness (Tb·Th), and trabecular number (Tb·N), confirms superior regenerative outcomes in the Mo-BGC group. * *p* < 0.05, ** *p* < 0.01 and *** *p* < 0.001 indicate significant differences between the indicated columns (two-way ANOVA) [92]. Reprinted with permission from Ref. [92]. Copyright 2022, ELSEVIER.

The immunoregulatory and regenerative performance of the Mo-BGC scaffold was evaluated in a clinically relevant critical-size periodontal defect model in canines. Defect-matched scaffolds demonstrated pronounced immunomodulatory activity, supporting stable macrophage regulation and promoting coordinated regeneration of multiple periodontal tissues (Figure 2a). To establish the surgical model, the first and third premolars (pre-M1 and pre-M3) in both mandibular quadrants were extracted before defect creation. Macrophage polarization within the defect region was assessed throughout a 28-day healing period using surface markers (CCR7 for M1 and CD206 for M2) and soluble mediators (iNOS for M1 and arginase for M2), in combination with CD68 labeling.

Immunofluorescence staining and quantitative analysis revealed significantly higher populations of Arg^+^/CD68^+^ and CD206^+^/CD68^+^ cells, corresponding to M2 macrophages, in tissues treated with Mo-BGC compared to undoped BGC scaffolds at both 7- and 28-day post-surgery (Figure 2b,c). In contrast, CCR7^+^/CD68^+^ and iNOS^+^/CD68^+^ M1 macrophages were more prevalent in the BGC group. Accordingly, Mo-BGC implantation resulted in a markedly reduced M1/M2 ratio, confirming its robust capacity to bias recruited macrophages toward a pro-regenerative M2 phenotype.

From a therapeutic standpoint, defects treated with both scaffold types achieved satisfactory soft tissue closure at 4 weeks and stable healing at 8 weeks post-surgery. However, mild gingival hyperemia and hemorrhage were observed at 1 week in sites receiving undoped BGC scaffolds. Longitudinal X-ray analysis further revealed that the presence of Mo ions slowed scaffold degradation during wound healing. Micro-CT evaluation demonstrated excellent anatomical matching between implants and defects at 1 week. By 8 weeks, substantial new bone formation was observed along the root surface and extending into the hollow channels of Mo-BGC scaffolds, accompanied by newly formed periodontal ligament tissue within radiolucent regions (Figure 2d).

Quantitative micro-CT analysis showed that Mo-BGC scaffolds exhibited a lower scaffold volume-to-total volume (SV/TV) ratio, indicating reduced degradation relative to BGC controls. In terms of osteogenesis, Mo-BGC treatment yielded the highest bone volume fraction (BV/TV) and trabecular number (Tb·N) among the three experimental groups (blank, BGC, and Mo-BGC) at 8 weeks post-surgery (*p* < 0.01). Trabecular thickness (Tb·Th) was also significantly increased compared with the blank control (*p* < 0.01; Figure 2e).

Collectively, this study provided the first demonstration of sustained immunomodulatory effects mediated by Mo ions released from a Mo-BGC scaffold in a large-animal periodontal defect model. Mechanistically, extracts from Mo-BGC powder were shown to regulate macrophage immunometabolism by shifting cellular energy production from glycolysis toward mitochondrial oxidative phosphorylation (OXPHOS), thereby directing macrophage polarization toward regenerative phenotypes. These findings highlight the pivotal role of metabolic signaling in Mo-mediated immune regulation within complex in vivo environments and offer valuable insights for the rational design of bioenergetic biomaterials in periodontal tissue engineering and regenerative medicine.

### 3.2. ROS Scavenging

Periodontitis is a chronic inflammatory condition that is closely linked to the presence of ROS. By eliminating ROS to reduce inflammation and manage the periodontal microenvironment, it may be possible to develop an effective treatment for periodontitis [93]. Exogenous antioxidants have been utilized to combat ROS and diminish inflammation in the treatment of periodontitis [94]. Various biologically active substances, such as natural enzymes and metal-based nanoenzymes, have been identified as effective ROS scavengers, demonstrating promising antioxidant capabilities [95]. However, the practical application of natural enzymes is often limited due to factors like the high costs associated with synthesis and purification, suboptimal reaction conditions, and inherent instability. Furthermore, the use of exogenous nanoagents or metal ions can introduce potential drawbacks, including adverse side effects and toxicity risks, which may raise additional biosafety concerns. Natural chemical compounds, rich in active antioxidant groups, are expected to serve as ROS scavengers in alternative treatments for diseases caused by oxidative stress [96].

Zhang et al. [97] developed a new artificial biocatalyst with a Pt-N active site, which imitates the natural enzyme catalase (CAT) to efficiently eliminate ROS in inflamed periodontal tissues and stem cell microenvironments. The biocatalyst, PtH-CN, equipped with multiple platinum clusters, demonstrated excellent ROS-elimination activities, effectively neutralizing H_2_O_2_, •O_2_^−^, •OH, and DPPH radicals (DPPH•). Among recently reported biocatalysts, PtH-CN exhibits exceptionally fast catalase-like activity, with a turnover number of 11.42 s^−1^. Additionally, PtH-CN can restore stem cell function and decrease the M1/M2 macrophage ratio, making it a safe and effective nanomedicine for treating periodontitis.

**Figure 3:**
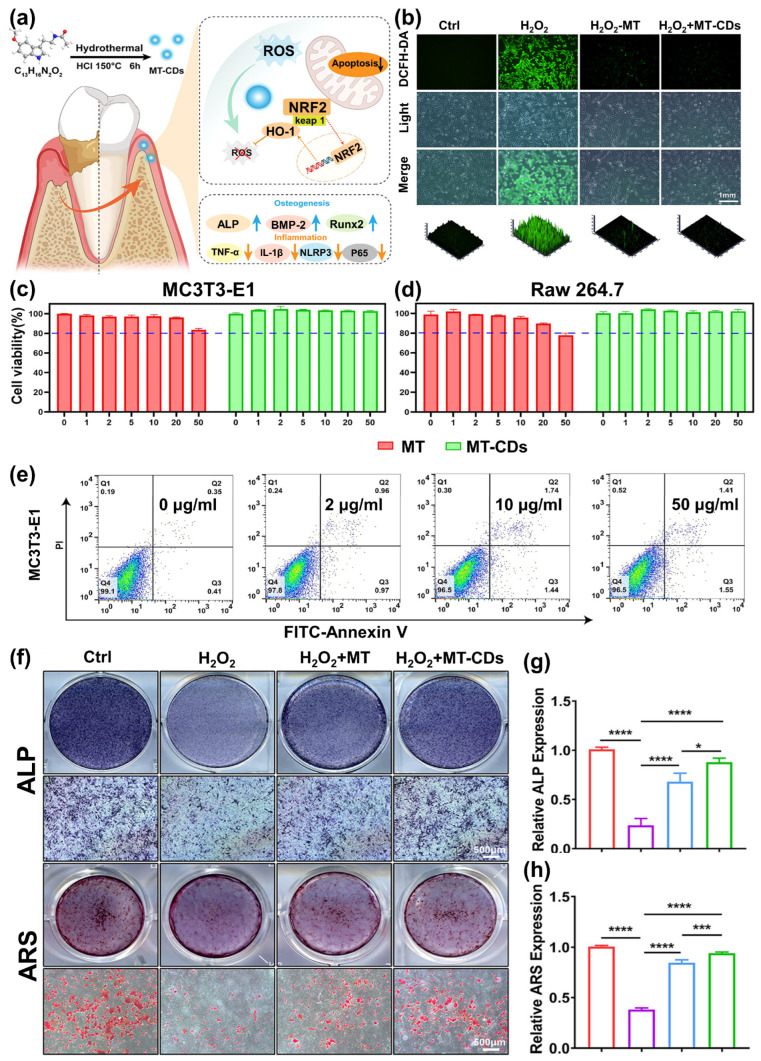
(**a**) Illustration of the design of MT-CDs and their anti-inflammatory and antioxidant properties for treating periodontal diseases and osteogenic activity. (**b**) Measurement of intracellular ROS levels using DCFH-DA detection. (**c**,**d**) Viability of MC3T3-E1 (**c**) and Raw 264.7 (**d**) cells after incubation with several concentrations of MT-CDs for 24 h. (**e**) Apoptosis analysis of MC3T3-E1 treated with MT-CDs. (**f**) The staining of ALP on day 7 and ARS on day 21. (**g**) Quantitative analysis of ALP activity. (**h**) Quantitative analysis of ARS mineral deposition, the data are presented as mean ± SD (*n* = 3). * means *p* < 0.05, *** means *p* < 0.001, **** means *p* < 0.0001. Abbreviations: Ctrl, control; MT, melatonin [98]. Reprinted with permission from Ref. [98]. Copyright 2024, AMERICAN CHEMICAL SOCIETY.

Xin et al. [98] synthesized melatonin-based carbon dots (MT-CDs) with water solubility using a simple hydrothermal method to enhance the therapeutic efficacy of periodontitis treatment. Compared to melatonin (MT), the prepared MT-CDs more effectively mitigated free radical-induced cellular damage, reduced inflammatory responses, and inhibited oxidative stress damage in vitro. In vivo, MT-CDs significantly inhibited alveolar bone resorption and reduced inflammation (Figure 3a). Notably, MT-CDs exhibited favorable biocompatibility, with negligible cytotoxicity and minimal impact on major organs. ROS can induce cellular damage and apoptosis via mitochondrial-mediated pathways. Specifically, oxidative stress can lead to mitochondrial dysfunction, reducing osteoblast activity and inducing osteoblast apoptosis, which can often be reversed by adding antioxidants. Based on green fluorescence intensity, it is evident that, compared to the control group, the intracellular ROS level significantly improved in the H_2_O_2_ group, while both the H_2_O_2_+MT and H_2_O_2_+MT-CDs groups showed a marked reduction (Figure 3b). The biosafety of MT-CDs was assessed in vivo and in vitro. To evaluate the in vitro cytotoxicity of MT-CDs, two cell lines, MC3T3-E1 and Raw 264.7, were treated with varying concentrations of MT-CDs for 24 and 72 h. The results were compared to those of MT at equivalent concentrations in both cell lines. The biocompatibility of MT-CDs was evaluated using the CCK-8 assay. For MC3T3-E1 cells treated with a dose of 50 μg/mL of MT, cell viability significantly decreased, whereas MT-CDs exhibited no substantial change in cell survival compared to the control group at the same concentration (Figure 3c). This suggested that, unlike MT, the cytotoxicity of MT-CDs was significantly reduced. Figure 3d shows that there was no significant reduction in Raw 264.7 viability upon exposure to MT-CDs at concentrations of 50 μg/mL compared to the control group. Meanwhile, with MT concentrations above 50 μg/mL, the survival rate of Raw 264.7 was already below 80%. The 72 h results aligned with those of 24 h, indicating that MT-CDs exhibited no noticeable cytotoxicity and demonstrated superior biocompatibility compared to MT. Moreover, the apoptosis of MC3T3-E1 remained nearly unchanged when treated with MT-CDs in dosages varying from 0 to 50 μg/mL (Figure 3e).

It has been reported that the enriched ROS and the resulting excessive inflammation at sites of bone injury impede the process of bone formation [99]. This process can contribute to the imbalance of bone remodeling, ultimately leading to bone loss. The moderate removal of excess ROS is important for managing the environment in periodontitis and creating favorable conditions for bone formation [100]. To explore the osteogenic potential under oxidative stress and inflammatory conditions, the protective properties of MT-CDs on MC3T3-E1cells under the H_2_O_2_-induced oxidative stress model and inflammation model induced by lipopolysaccharide (LPS) were also investigated. To investigate the osteoblastic effects promoted by MT-CDs under oxidative stress conditions, ALP and ARS assays were conducted. ALP activity is positively correlated with the area of staining (blue). The results suggested that, in comparison to the H_2_O_2_ group, both the H_2_O_2_+MT and H_2_O_2_+MT-CDs groups substantially enhanced the ALP expression level (Figure 3f), and the semiquantitative analysis indicates that the MT-CDs had a more significant effect than the MT (Figure 3g). Subsequently, extracellular calcification was observed by using an alizarin red stain. The quantification of stained spots was performed to assess the mineralization activity in each group. As seen from Figure 3f, the results of alizarin red stain confirmed those of ARS staining. The positive control showed a large number of red nodules, indicating that calcium deposition was caused by osteogenic differentiation. The group treated with H_2_O_2_ exhibited a reduced number of spots as a result of the suppression of ROS. Meaningfully, this process was reversed in the H_2_O_2_+MT and H_2_O_2_+MT-CDs groups. The alizarin red staining and its semiquantitative analysis further demonstrated that MT-CDs have a more significant advantage in promoting osteogenesis under ROS conditions than MT (Figure 3h).

### 3.3. Controlled Release Systems

New developments in localized drug delivery systems have greatly enhanced the treatment of periodontitis. By embedding multiple bioactive agents into polymeric scaffolds, especially through nanocarrier-based systems, it is possible to achieve controlled and sustained release at the site of infection [101]. Lipid-based nanoparticles (LNPs) are particularly effective in enhancing the delivery of poorly soluble compounds by improving their stability, bioavailability, and cellular uptake [102]. When incorporated into hydrogel or membrane-based platforms, these systems can deliver a combination of antibacterial, anti-inflammatory, and regenerative agents simultaneously. This comprehensive approach not only extends drug retention time but also ensures targeted delivery, overcoming the limitations of conventional treatments [103]. Such platforms show strong potential to promote both soft tissue healing and bone regeneration, marking a promising direction in the development of effective, locally acting therapeutics for periodontal disease.

**Figure 4:**
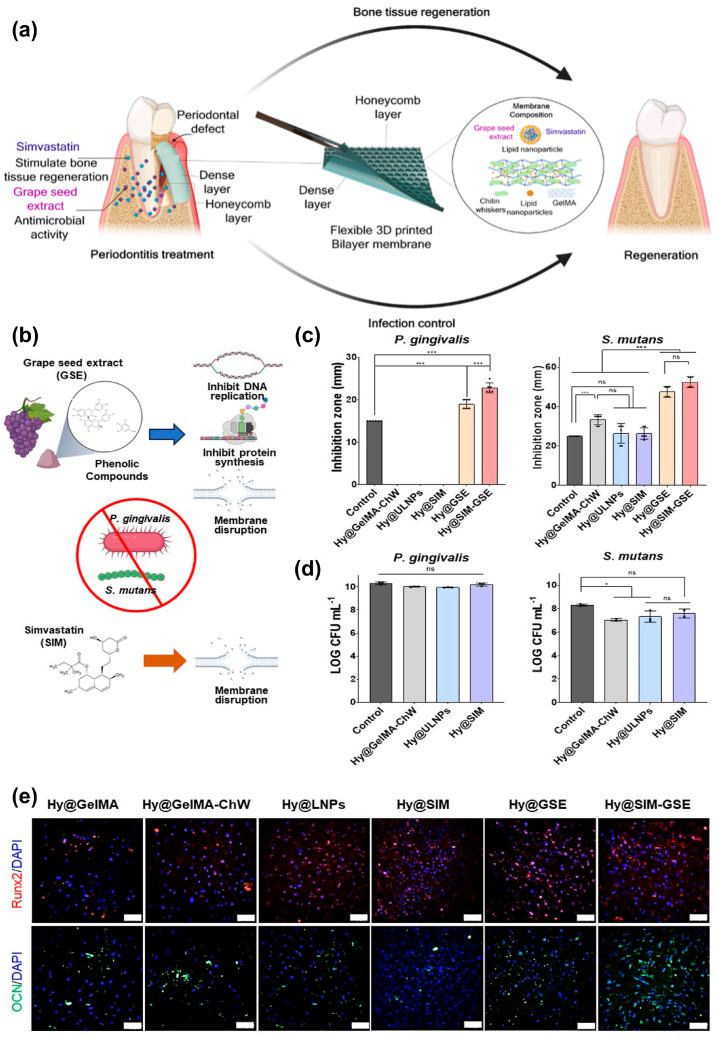
(**a**) A diagram showing the fabrication process of 3D-printed bi-layer constructs incorporating nanomaterial-based composite hydrogels for treating periodontitis and bone regeneration. (**b**) Antimicrobial mechanisms of GSE and SIM against *P. gingivalis* and *S. mutans*. (**c**) Inhibition halo analysis values and (**d**) CFU counts after exposing bacterial strains to different composite hydrogel formulations (ns: not significant, * *p* < 0.05, and *** *p* < 0.001; one-way ANOVA with Tukey’s multiple comparisons test). (**e**) Immunofluorescence staining of the osteogenic markers Runx2 and OCN after hMSC culture on composite hydrogel surfaces on day 21 in osteogenic differentiation media [104]. Reprinted with permission from Ref. [104]. Copyright 2024, AMERICAN CHEMICAL SOCIETY.

Santos et al. [104] developed a multifunctional 3D-printed bilayered composite hydrogel that serves as a dual drug delivery system for treating periodontitis and promoting bone regeneration (Figure 4a). This approach involved loading grape seed extract (GSE) and simvastatin (SIM) into LNPs, which, together with chitosan whiskers (ChW), were incorporated into a gelatin–methacryloyl (GelMA)-based gel matrix. This composite hydrogel ink was used in the direct ink writing (DIW) 3D printing process to create bilayered membranes with honeycomb and dense layers, mimicking the hierarchical structure of the periodontium. The dense layer within the scaffold was designed to isolate the bone defect from the surrounding fibrous connective tissue. The resulting membranes were characterized by their mechanical properties, drug-release profile, susceptibility to enzymatic degradation, in vitro antimicrobial activity, biocompatibility, and capacity to stimulate osteogenesis. The efficacy of the engineered bilayered construct against both Gram-positive *S. mutans* and Gram-negative *P. gingivalis* was evaluated, as these bacterial strains are recognized for causing chronic and aggressive forms of periodontitis. The antimicrobial activity of GSE is primarily attributed to its phenolic compounds, which effectively inhibit bacterial DNA replication and protein synthesis while also causing membrane disruption (Figure 4b). Since GSE is derived from natural sources, it serves as an alternative or complementary treatment to synthetic antibiotics. The antimicrobial activity of the membranes was investigated using both inhibition halo analysis and colony-forming unit (CFU) counts (Figure 4c,d). Only hydrogels containing GSE effectively inhibited the growth of the tested bacteria. Notably, formulations that included both drugs demonstrated larger bacterial inhibition zones than those containing only GSE. Furthermore, nanocomposite hydrogels provided scaffold function for the regeneration of periodontal tissue. In vivo studies confirmed their efficacy in enhancing alveolar bone regeneration and reducing inflammation in a periodontal defect model. After human mesenchymal stem cells (hMSCs) were cultured on the surface of the hydrogels for 14 and 21 days, both in osteogenic and nonosteogenic media, osteogenesis was corroborated by analyzing the levels of in vitro expression of bone-specific markers, including osteocalcin (OCN) and runt-related transcription factor 2 (Runx2). Runx2, a marker of early osteogenesis, and osteocalcin, a non-collagenous protein deposited in mineralized adult bones by osteoblasts, were used to evaluate osteogenic differentiation. Immunofluorescence images for assessing the expression of OCN and runx2 are presented in Figure 4e. Red staining corresponds to the expression of Runx2, while green staining is related to OCN expression. These results indicate a higher expression of Runx2 and osteocalcin markers in cells incubated with Hy@SIM-GSE when exposed to the osteogenic medium compared to other hydrogel formulations incubated under the same conditions. These results suggest that including SIM and GSE drugs in hydrogels may boost osteogenic differentiation of hMSCs.

The proposed bilayered membrane holds the potential for periodontal regeneration owing to its features and advantages. It is composed of low-cost biocompatible materials, which makes it a more accessible and affordable treatment option for patients. Moreover, the hydrogel was designed to simultaneously promote bone tissue regeneration and prevent bacterial contamination, which is beneficial for treating periodontitis. These structures and features make the proposed drug delivery system an effective and versatile approach for periodontal regeneration.

### 3.4. Combinatory ROS Scavenging and Gas Therapy

In the pathogenesis of chronic periodontitis, elevated levels of ROS play a destructive role by directly attacking essential biomolecules such as DNA, lipids, and proteins. This oxidative stress disrupts normal cellular function, hinders cellular proliferation, and results in progressive deterioration of periodontal tissues [105]. Additionally, ROS overproduction acts as a key initiator of inflammatory signaling cascades, thereby intensifying the inflammatory environment. One prominent example is the activation of the NF-κB pathway, which drives the transcription of genes responsible for promoting macrophage polarization toward the pro-inflammatory M1 phenotype. This polarization enhances the secretion of cytokines, chemokines, and other mediators that sustain chronic inflammation [106]. Concurrently, the Nrf2, a critical transcription factor that orchestrates the cellular antioxidant defense system, is downregulated in oxidative environments. Nrf2 normally upregulates antioxidant gene expression to mitigate oxidative damage; however, excessive ROS suppresses this protective mechanism, preventing the transition of macrophages to the anti-inflammatory M2 phenotype [107]. This dysregulation in macrophage polarization leads to an increased M1/M2 ratio, creating an unfavorable environment for periodontal regeneration [108]. Moreover, sustained oxidative stress impairs the regenerative capacity of stem cells and enhances osteoclastic activity, ultimately contributing to alveolar bone resorption [109]. To address these interconnected pathological mechanisms, there is a growing demand for multifunctional therapeutic platforms capable of scavenging ROS, restoring macrophage homeostasis, promoting osteogenesis, and stimulating bone regeneration. In this context, several types of bioactive agents, such as natural polyphenols, metal-based nanozymes, and enzymatic antioxidants, have been employed as effective ROS scavengers with promising results in periodontal therapy [110]. These materials exhibit favorable antioxidant and anti-inflammatory properties. However, these materials often face challenges such as labor-intensive synthesis or modification processes and safety concerns, which limit their further application [111,112].

**Figure 5:**
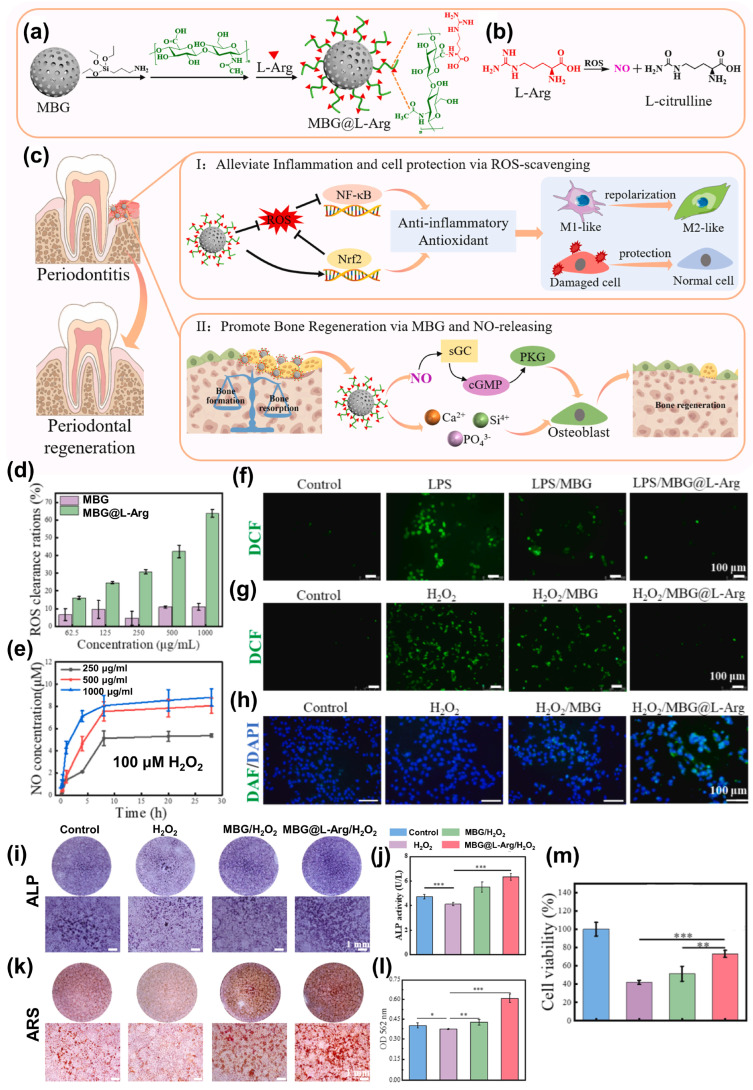
(**a**) Diagram showing the preparation of MBG@L-Arg. (**b**) MBG@L-Arg action mechanism for ROS scavenging and NO release. (**c**) Diagrammatic representation of the MBG@L-Arg application against periodontitis and bone regeneration. (**d**) MBG@L-Arg ROS scavenging capacity. (**e**) MBG@L-Arg NO release capacity. (**f**) RAW264.7 cells and (**g**) MC3T3-E1 cells images stained with DCFH-DA. (**h**) MC3T3-E1 cells images stained with DAF-FM DA. (**i**,**j**) ALP staining images and activity on day 7. (**k**,**l**) ARS staining images and quantitative analysis on day 14. (**m**) Cell viability of MC3T3-E1 cells, the data are presented as mean ± SD (*n* = 5). * means *p* < 0.05, ** means *p* < 0.01, *** means *p* < 0.001. Reprinted from Ref. [113].

Yao et al. [113] developed a biocompatible L-arginine-modified mesoporous bioactive glass (MBG@L-Arg) through a straightforward and environmentally sustainable process. The synthesis began with the preparation of mesoporous bioactive glass (MBG), which was functionalized with 3-aminopropyltrimethoxysilane (APTES). Hyaluronic acid (HA) was then grafted onto the APTES-modified MBG, introducing abundant carboxyl groups. Finally, L-arginine (L-Arg) was covalently attached via amide bond formation between HA and the MBG surface (Figure 5a). This hybrid material demonstrated excellent capabilities for ROS scavenging, NO release, and inflammation modulation, effectively mitigating periodontal tissue damage and alveolar bone loss. In cellular assays, MBG@L-Arg showed significant NO generation and strong ROS neutralization, protecting cell viability under oxidative conditions. It also induced a phenotypic shift in macrophages from pro-inflammatory M1 to anti-inflammatory M2, while promoting osteogenesis in both MC3T3-E1 preosteoblasts and human periodontal ligament stem cells (hPDLSCs). Mechanistically, MBG@L-Arg inhibited the NF-κB signaling pathway while activating the antioxidant Nrf2 pathway and the NO-mediated PKG signaling axis, collectively enhancing tissue repair and bone regeneration (Figure 5b,c). MBG alone exhibited limited ROS scavenging (<10% at 1000 μg/mL). In contrast, MBG@L-Arg achieved 30.8% ROS elimination at 250 μg/mL and 63.8% at 1000 μg/mL (Figure 5d). NO levels released by MBG@L-Arg in response to 100 μmol/L H_2_O_2_ reached 5.4, 7.9, and 8.8 μmol/L at 250, 500, and 1000 μg/mL, respectively (Figure 5e), underscoring its dual function as a potent antioxidant and NO donor. The electron-donating guanidino groups of L-Arg terminated free radical chains, while guanidine nitrogen was oxidized intracellularly to generate NO, further promoting healing.

Fluorescence microscopy revealed efficient uptake of MBG@L-Arg by macrophages, likely due to HA-CD44 interaction. Intracellular ROS reduction was visualized by DCFH-DA staining. In RAW264.7 cells, LPS stimulation resulted in 71.2 ± 2.7% ROS-positive cells, similar to the MBG group (70.1 ± 6.4%), suggesting MBG alone lacks antioxidant capacity. However, MBG@L-Arg reduced this value to 4.8 ± 2.2% (Figure 5f). Similarly, in MC3T3-E1 cells exposed to H_2_O_2_, ROS positivity dropped from 91.3 ± 6.0% to 6.7 ± 2.6% following MBG@L-Arg treatment (Figure 5g), nearly matching the control group. NO generation within MC3T3-E1 cells was assessed using the DAF-FM DA probe. Minimal green fluorescence was observed in the control and LPS groups, while the MBG group displayed a weak signal, possibly from Ca^2+^-mediated NO synthase activation. In contrast, MBG@L-Arg-treated cells exhibited strong fluorescence, indicating substantial NO release (Figure 5h). To test osteogenesis under oxidative stress, MC3T3-E1 cells were treated with H_2_O_2_. The H_2_O_2_ group showed reduced ALP activity, reflecting impaired osteogenic differentiation. MBG treatment restored some ALP levels, but MBG@L-Arg induced robust ALP expression, visualized as widespread purple staining (Figure 5i,j). Alizarin red S staining confirmed mineralized nodule formation, which was significantly enhanced in the MBG@L-Arg group, with the highest absorbance values among all samples (Figure 5k,l). Finally, MBG@L-Arg demonstrated protective effects against ROS-induced cytotoxicity. While MBG-treated cells showed 51.2 ± 8.1% viability, MBG@L-Arg pretreatment improved this to 73.1 ± 3.9% under H_2_O_2_ challenge (Figure 5m), emphasizing its role in maintaining cell survival under oxidative stress.

Non-bacterial dental infections, often characterized by chronic inflammation and oxidative stress rather than direct microbial invasion, require therapeutic strategies focused on immune modulation and tissue repair. Immunotherapy offers a means to regulate host responses and promote regeneration, while advanced drug delivery systems allow localized and sustained action with reduced systemic toxicity. ROS scavenging therapies mitigate oxidative damage and create a favorable microenvironment for stem cell survival and osteogenesis, whereas gas-based therapies introduce bioactive signals that simultaneously control inflammation and stimulate vascularization and bone remodeling. Although each strategy faces hurdles such as controlling release kinetics, avoiding immune over-suppression, or ensuring biosafety, their integration with smart biomaterials presents an opportunity to advance beyond symptomatic care toward targeted, regenerative management of oral diseases. The potential benefits and clinical outlook of these strategies are detailed in Table 1.

## 4. Multimodal Strategy for Dental Infection Management

Addressing periodontitis through a combined treatment approach for both bacterial and nonbacterial dental infections offers a comprehensive solution to the multifaceted challenges of this condition [114]. By integrating antibacterial strategies, such as the use of nanozymes with potent ROS-scavenging capabilities, with bone regeneration techniques like injectable hydrogels, this integrated method effectively eradicates infectious pathogens and promotes the restoration of damaged periodontal tissues [115,116]. This dual-action approach enhances overall therapeutic efficacy by ensuring that both the bacterial infection and the associated inflammatory response are managed effectively, while simultaneously supporting the regeneration of alveolar bone and periodontal ligament. This holistic treatment strategy holds significant promise for improving clinical outcomes and reducing the recurrence rates of periodontitis, ultimately leading to better long-term oral health.

Xu et al. [117] developed a hydrogel system called TM/BHT/CuTA, which combines triglycerol monostearate/2,6-di-tert-butyl-4-methylphenol (TM/BHT) hydrogel with copper tannic acid coordination NSs (CuTA NSs). This system leverages the negative charge of TM/BHT/CuTA to adhere to cationic inflammation sites through electrostatic interactions. It also responds to the elevated levels of matrix metalloproteinase (MMP) in periodontitis by hydrolyzing and releasing the CuTA nanozyme on demand. Both antiplaque and antibacterial activities were demonstrated by the released CuTA nanozyme. By imitating the cascade reactions of SOD and catalase, it functioned as a metal-phenolic nanozyme that efficiently scavenged various ROS. Additionally, through the Nrf2/NF-κB pathway, the CuTA nanozyme altered macrophage polarization from the pro-inflammatory M1 phenotype to the anti-inflammatory M2 phenotype. This alteration diminished inflammation and sped up tissue regeneration in periodontitis by increasing anti-inflammatory cytokines, decreasing pro-inflammatory cytokines, and encouraging the expression of osteogenic genes.

Zhao et al. [118] created a glycopeptide hydrogel, referred to as GRWgel, designed to facilitate alveolar bone resorption through sequential RANKL-blocking and antimicrobial activities for the treatment of periodontitis. GRWgel exhibited an ECM-like porous and fibrous microstructure that served as a scaffold for cell differentiation and proliferation. It also possessed several beneficial properties, including self-healing capabilities, injectability, and adequate cell viability. Upon in situ inclusion, the hydrogel rapidly released MH in the initial phase, demonstrating an effective antibacterial effect against biofilms in deep periodontal pockets. Moreover, MH/GRWgel showed elevated specific binding efficacy with RANKL, inhibiting the maturation of osteoclasts by blocking the RANK/RANKL interaction and increasing osteogenic differentiation. This dual mechanism simultaneously regulates bone homeostasis. In a rat model of periodontitis, MH/GRWgel substantially curtailed the development of periodontitis through inhibition of alveolar bone resorption, antibacterial activity, and promotion of bone regeneration. These effects were superior to those achieved with a commercial gel.

**Figure 6:**
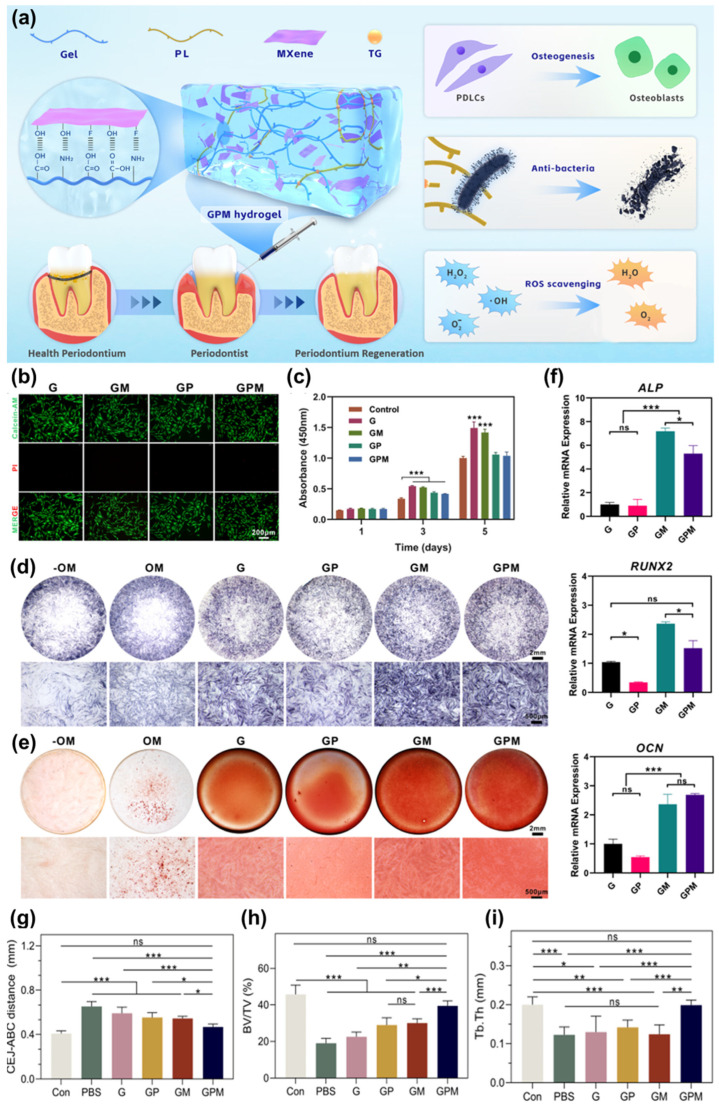
(**a**) Illustration of the design of injectable GPM and its application in treating periodontal disease. (**b**) Live/dead staining of hPDLCs seeded on G and GPM hydrogels after 48 h. (**c**) Cell proliferation of hPDLCs cultured with extraction media from different hydrogels. (**d**) ALP staining of hPDLCs treated with hydrogels at 5 days. (**e**) ARS staining of hPDLCs co-cultured after 14 days with different hydrogels. (**f**) Relative expression of osteogenesis-related genes in hPDLCs co-cultured with hydrogels. Quantitative analysis of the bone-related parameters of the M2, including (**g**) CEJ-ABC distance, (**h**) BV/TV, and (**i**) Tb.Th from the micro-CT images, Data are presented as the mean ± SD, *n* = 5. ns: not significant, * *p* < 0.05; ** *p* < 0.01; *** *p* < 0.001 [119]. Reprinted with permission from Ref. [119], Copyright 2024, AMERICAN CHEMICAL SOCIETY.

Yu et al. [119] developed an injectable hydrogel to treat periodontitis, effectively minimizing bacterial infection and inflammation while supporting bone formation. The hydrogel was based on gelatin and enhanced with inorganic MXene NSs and PL peptides (Figure 6a). The addition of MXene NSs significantly boosted the hydrogel’s anti-inflammatory properties and enhanced its osteoconductivity, making it an effective scaffold for bone regeneration. Incorporating PL peptides provides the hydrogel with potent antibacterial capabilities, particularly against *P. gingivalis*. The porous structure of hydrogel ensured suitable biodegradability and wet adhesive properties, which are essential for its application in periodontal defects. These features made the hydrogel a perfect scaffold for cell proliferation and differentiation, promoting the alveolar bone tissue regeneration. In vitro studies demonstrated the excellent biocompatibility of hydrogel with human periodontal ligament cells (hPDLCs) (Figure 6b,c). To evaluate the hydrogel’s effects on osteogenic differentiation, ALP, ARS, and qRT-PCR assays were conducted. After 5 days of treatment, the GM and GPM groups exhibited significantly greater ALP activity in contrast to the control, G, and GP groups (Figure 6d). This indicates that the addition of MXene NSs significantly enhances the osteoinductivity of the hydrogels. The ARS staining revealed deeper redness in the MXene-modified hydrogels, with the highest mineralization level observed in the GM group (Figure 6e). Consistently, qRT-PCR analysis showed elevated expression of osteogenic-related genes (RUNX2, ALP, and OCN) in the GM and GPM groups (Figure 6f). In vivo studies were conducted using a rat model of periodontitis. Histopathological assessments of the heart, liver, spleen, lungs, and kidneys confirmed the hydrogel’s biocompatibility. The PBS group showed a significant reduction in alveolar bone height. The application of GM and GP hydrogels restored periodontal tissue to some extent, indicating that the antibacterial PL and the bioactive, ROS-scavenging MXene played positive roles in reducing inflammation and improving bone-healing ability. The injection of the gelatin-based hydrogels remarkably alleviated alveolar bone loss near the maxillary second molars. The specific values of the CEJ-ABC distance in the control, PBS, G, GP, GM, and GPM groups were 0.41 ± 0.03, 0.69 ± 0.06, 0.59 ± 0.06, 0.55 ± 0.04, 0.55 ± 0.02, and 0.46 ± 0.03 mm, respectively (Figure 6g). The GM and GP hydrogels promoted alveolar bone regeneration compared to the G gels. There was no significant difference in the CEJ-ABC distance between the GP- and GM-treated groups. In particular, the CEJ-ABC distance of the GPM-treated groups nearly recovered to that of the normal group, indicating positive effects of the GPM hydrogel on alveolar bone regeneration. In addition, the GPM-treated group showed the highest BV/TV and Tb. Th values of 39.49 ± 2.64% and 0.20 ± 0.01 mm, respectively, were comparable to those of the healthy group (45.72 ± 5.16% and 0.20 ± 0.02 mm, respectively) (Figure 6h,i). The combination of antibacterial PL and bioactive MXene in the GPM gels provided the most favorable microenvironment for periodontal healing, as evidenced by the lowest IL-1β expression and the strongest OCN staining intensity. To facilitate effective regeneration, nanomaterials must be engineered with specific degradation rates and release profiles tailored to their intended application, which are detailed in Table 2.

## 5. Future Challenges and Perspectives

Despite the remarkable advances in our understanding of the etiology and progression of periodontal disease, the translation of next-generation therapeutic approaches into consistent clinical outcomes remains a formidable challenge. The oral cavity represents a highly dynamic microenvironment, where constant mechanical forces, fluctuating pH, enzymatic activity, and complex microbial communities continuously test the durability and efficacy of novel interventions. While nanotechnology, smart biomaterials, and targeted delivery strategies have introduced viable alternatives to conventional antibiotics, their clinical adoption is still hindered by multifaceted biological, material, and regulatory barriers. This section highlights the key hurdles and outlines future directions that demand interdisciplinary solutions to achieve sustainable clinical success.

### 5.1. Advanced Coating Materials for Periodontal and Implant Interfaces

A critical challenge in periodontitis-associated tooth loss and implant therapy is the prevention of peri-implant inflammation and infection arising from biofilm formation and dysregulated host responses at the implant–tissue interface. Advanced surface-engineered coatings have emerged as promising strategies to mitigate bacterial colonization while modulating local inflammatory pathways. Nanostructured ceria coatings, piezoelectric films, and layer-by-layer drug-eluting surfaces have demonstrated the ability to suppress peri-implant biofilms and attenuate inflammatory signaling in preclinical models. Nevertheless, maintaining durable bioactivity under clinically relevant conditions, including salivary flow, masticatory forces, and recurrent microbial exposure, remains a significant hurdle. Future efforts must prioritize coatings capable of long-term stability, biofilm resistance, and adaptive immune modulation tailored to the periodontal microenvironment to ensure sustained peri-implant tissue integration and function.

### 5.2. Next-Generation Drug Delivery Systems

Localized and controlled drug delivery remains central to the management of periodontitis, where sustained therapeutic concentrations are required within periodontal pockets and inflamed tissues. Chitosan-based carriers, in situ-forming depots generated via solvent-induced precipitation, and microsphere-based delivery systems have shown promise in achieving prolonged antimicrobial and anti-inflammatory release at disease sites. Increasingly, multifunctional platforms integrating antibacterial agents with host-modulatory or antioxidant therapeutics within biodegradable matrices have demonstrated efficacy in addressing both microbial dysbiosis and inflammation-driven tissue destruction. Despite these advances, challenges related to formulation reproducibility, regulatory approval, and scalable manufacturing continue to impede clinical translation. Future research must focus on optimizing delivery kinetics, biocompatibility, and degradation profiles while ensuring compatibility with existing periodontal treatment workflows.

### 5.3. Regenerative Platforms for Bone and Periodontal Repair

Long-term management of dental infections also depends on restoring the structural and functional integrity of damaged tissues. Biomaterials such as bioactive scaffolds, injectable hydrogels, and adhesive composites are being engineered to support periodontal and alveolar bone regeneration. Incorporating osteoinductive microspheres, stem cell recruitment cues, and vascularization factors into these platforms represents an important step forward. However, translating these regenerative strategies into clinical dentistry requires a deeper understanding of immunomodulation, redox balance, and host-biomaterial interactions to ensure predictable healing.

### 5.4. Functionalization of 3D-Printed Dental Devices

Additive manufacturing has revolutionized the design of patient-specific dental prosthetics and scaffolds. However, the incorporation of antibacterial and anti-inflammatory functionalities into 3D-printed constructs is still at an early stage. Embedding nanoparticles, antimicrobial peptides, or drug-releasing adhesives within 3D-printed matrices may offer a route to personalized, infection-resistant devices. The main challenge is maintaining mechanical stability and biocompatibility while ensuring controlled release in the variable oral environment.

### 5.5. Integration of Digital and Intelligent Technologies

Artificial intelligence holds immense promise for enhancing diagnostic accuracy, optimizing biomaterial design, and guiding dynamic treatment decisions. AI-driven models could eventually personalize therapeutic interventions according to a patient’s microbiome, disease state, and healing trajectory. Nonetheless, its integration into clinical practice is in its infancy, with challenges in data standardization, clinical validation, and ethical governance. Coupling AI-based monitoring with bioresponsive materials represents an exciting but underexplored avenue.

### 5.6. Overarching Barriers and Future Directions

Several overarching hurdles continue to hinder the translation of these advanced therapies. The heterogeneity of oral microbiota across individuals highlights the need for adaptable, patient-specific platforms rather than universal solutions. The dynamic oral environment requires highly stable, multifunctional materials capable of sustaining efficacy under fluctuating pH, salivary enzymes, and mechanical load. Furthermore, combinatory therapies, though promising in preclinical studies, face challenges in scalability, regulatory approval, and safety validation, particularly regarding biodistribution and degradation byproducts.

### 5.7. Pellicle-Driven Barriers to Targeted Oral Therapies

In the complex environment of the oral cavity, the efficacy of therapeutic nanomaterials is significantly influenced by the immediate adsorption of salivary proteins and glycoproteins onto oral surfaces, forming the “acquired pellicle,” which acts as a critical mediator for microbial adhesion and serves as the primary interface for biomaterial-host interactions. Consequently, a rational design of periodontal therapies must account for this protein layer to ensure sustained bioactivity and targeted delivery within the challenging oral milieu.

Future research should prioritize the design of bioresponsive nanomaterials that incorporate immunoregulatory and redox-balancing properties, alongside the development of scaffolds capable of promoting stem cell recruitment, angiogenesis, and bone regeneration. Equally important is the integration of real-time, AI-driven monitoring systems to enable adaptive therapies tailored to disease progression. These advancements must be supported by well-structured, long-term clinical trials that thoroughly evaluate efficacy, safety, patient compliance, and cost-effectiveness. Achieving these goals will require close collaboration across material science, microbiology, and digital medicine to translate innovative concepts into reliable, personalized treatments for dental infections.

## 6. Conclusions

Periodontitis is a complex, chronic inflammatory disease driven by microbial dysbiosis, sustained oxidative stress, and an imbalanced host immune response, ultimately resulting in irreversible damage to periodontal tissues. This multifactorial pathology necessitates therapeutic strategies that extend beyond conventional antimicrobial approaches toward interventions capable of regulating inflammation and promoting tissue regeneration. In this review, we have highlighted nanobiotechnology-based platforms that address these interconnected processes through immune modulation, redox regulation, and bioactive material design.

Emerging systems such as ROS-scavenging hydrogels, gas-releasing therapeutic platforms, and multifunctional scaffolds demonstrate the potential to simultaneously suppress pathological inflammation, enhance angiogenesis, and support osteogenic regeneration within periodontal defects. These approaches represent a shift from symptomatic disease management toward biologically informed regeneration tailored to the periodontal microenvironment.

Despite promising preclinical outcomes, significant challenges remain for clinical translation, including long-term biosafety, material stability in the oral environment, and the need for standardized evaluation frameworks that reflect clinical reality. Future progress will depend on the development of multifunctional biomaterials that can adapt to the dynamic periodontal niche while maintaining therapeutic precision and regenerative efficacy. Continued interdisciplinary collaboration across materials science, immunology, and periodontal research will be essential to advance these technologies from experimental platforms toward clinically viable solutions for periodontal regeneration.

## Figures and Tables

**Table 1:** Comparative analysis of host-directed therapeutic strategies for dental health care management.

Therapeutic Strategy	Advantages	Disadvantages	Clinical Potential
Immunotherapy	Targets host immune pathways rather than pathogens. Can rebalance inflammatory microenvironment (e.g., M1 → M2 macrophage shift). Supports tissue regeneration while reducing chronic inflammation.	Risk of over-suppression leading to secondary infection. Patient-to-patient variability in immune response. Requires precise dosing and monitoring.	Promising in periodontal and pulpal inflammation; still in preclinical and early translational exploration.
Drug Delivery Systems	Localized and sustained release at the disease site. Minimizes systemic side effects. Can be integrated with scaffolds, hydrogels, or nanocarriers.	Risk of burst release or insufficient drug retention. Complex formulation may raise production costs. Potential for limited penetration in dense tissues.	Widely studied; several systems (e.g., hydrogels, microspheres) have shown translational potential for regenerative dentistry.
ROS Scavenging Therapy	Neutralizes oxidative stress in inflamed tissues. Protects stem cells and enhances bone regeneration. Can synergize with other regenerative strategies.	Over-scavenging may impair physiological ROS signalling. Efficacy depends on local ROS levels. Long-term biosafety remains to be validated.	Strong preclinical evidence in periodontitis and pulpitis; likely to enter clinical testing with multifunctional biomaterials.
Gas Therapy (e.g., NO, H_2_S, CO)	Gasotransmitters regulate inflammation, angiogenesis, and bone metabolism. Dual role: antimicrobial action plus tissue regeneration. Can be engineered into smart release platforms.	Difficult to control dosage and release kinetics. Some gases may have systemic toxicity at high levels. Delivery systems are technically challenging.	Emerging field, promising for periodontal and alveolar bone regeneration, but still at an early experimental stage.

**Table 2:** Rational design parameters for nanomaterials in periodontal tissue engineering.

Strategy	Target Application	Key Nanomaterials	Degradation and Release Profile	Functional Requirements and Design	References
Immunotherapy	Modulation of M1/M2 macrophage polarization	Gold NPs, Chitosan-based NPs, Functionalized Mesoporous Silica	Slow/Controlled: Needs to persist in the inflamed site to maintain the anti-inflammatory microenvironment.	Surface charge and Ligands: Designed to interact with immune cell receptors to suppress pro-inflammatory cytokines.	[120,121]
Drug Delivery Systems	Delivery of Growth Factors (BMP-2) or Antibiotics	PLGA NPs, Liposomes, Hydrogel Nanocomposites	Stimuli-Responsive: Degradation triggered by local pH changes or enzymes (MMPs) to ensure release only at the infection site.	Biocompatibility: High surface-to-volume ratio for maximum loading of osteogenic or antimicrobial agents.	[122,123,124]
ROS Scavenging Therapy	Neutralizing oxidative stress in periodontal pockets	Ceria (CeO_2_) NPs, Lignin NPs, Carbon Dots	Catalytic/Persistent: Material should act as a “nanozyme” with a long half-life to provide continuous antioxidant protection.	Porosity: High surface area to maximize contact with reactive oxygen species; prevents cell death in osteoblasts.	[125,126]
Gas Therapy (NO, H_2_S, CO)	Angiogenesis and Antimicrobial action	Metal–Organic Frameworks (MOFs), Hollow Mesoporous Silica	Burst followed by Sustained: Rapid initial release for biofilm disruption; slow release for promoting blood vessel formation.	Site-Specific: Gas precursors must be encapsulated to prevent premature systemic release; targeted at deep periodontal pockets.	[127]

## Data Availability

No new data were created or analyzed in this study. Data sharing is not applicable to this article.

## References

[1] Öçbe M., Çelebi E., Öçbe Ç.B. (2025). An Overlooked Connection: Oral Health Status in Patients with Chronic Diseases. BMC Oral Health.

[2] Zhu M., Hao C., Zou T., Jiang S., Wu B. (2025). Phage Therapy as an Alternative Strategy for Oral Bacterial Infections: A Systematic Review. BMC Oral Health.

[3] Garay-Sarmiento M., Yayci A., Rutsch Y., El Kadaoui H., Apelt S., Englert J., Boes A., Kohse M., Jakob F., Bergs T. (2025). Structure Protects Function: A Multilevel Engineered Surface Modification Renders the Surface of Titanium Dental Implants Resistant to Bacterial Colonization. ACS Appl. Mater. Interfaces.

[4] Kowalski J., La Rosa G.R.M., Di Stefano A., Gangi D., Sahni V., Yilmaz H.G., Fala V., Górska R., Ludovichetti F.S., Amaliya A. (2025). Navigating the Dual Burden of Dental and Periodontal Care in Individuals Who Also Smoke: An Expert Review. J. Dent..

[5] Tao J., Sun Y., Wang G., Sun J., Dong S., Ding J. (2025). Advanced Biomaterials for Targeting Mature Biofilms in Periodontitis Therapy. Bioact. Mater..

[6] Tsuchida S., Umemura H., Iizuka K., Yamamoto H., Shimazaki I., Shikata E., Nakayama T. (2024). Recent Findings on Metabolomics and the Microbiome of Oral Bacteria Involved in Dental Caries and Periodontal Disease. World J. Microbiol. Biotechnol..

[7] Romandini P., Marruganti C., Romandini W.G., Sanz M., Grandini S., Romandini M. (2024). Are Periodontitis and Dental Caries Associated? A Systematic Review with Meta-analyses. J. Clin. Periodontol..

[8] Stangvaltaite-Mouhat L., Skudutyte-Rysstad R., Ko H., Stankeviciene I., Aleksejuniene J., Puriene A. (2024). Co-Occurrence of Dental Caries and Periodontitis: Multilevel Modelling Approach. BMC Oral Health.

[9] Heitz-Mayfield L.J.A. (2024). Conventional Diagnostic Criteria for Periodontal Diseases (Plaque-Induced Gingivitis and Periodontitis). Periodontology 2000.

[10] Chu F., Wu H., Li C., Qiu W., Zang L., Wu D., Shao J., Wang T., Wang C. (2025). Transcriptomics Analysis Reveals the Effect of *Pulsatilla* Decoction Butanol Extract on Endoplasmic Reticulum and Peroxisome Function of *Candida albicans* in Hyphal State. J. Ethnopharmacol..

[11] Zheng Y., Wang Z., Weng Y., Sitosari H., He Y., Zhang X., Shiotsu N., Fukuhara Y., Ikegame M., Okamura H. (2025). Gingipain Regulates Isoform Switches of PD-L1 in Macrophages Infected with Porphyromonas Gingivalis. Sci. Rep..

[12] Thayumanavan T., Harish B.S., Subashkumar R., Shanmugapriya K., Karthik V. (2025). Streptococcus Mutans Biofilms in the Establishment of Dental Caries: A Review. 3 Biotech.

[13] Dakalbab S., Hamdy R., Holigová P., Abuzaid E.J., Abu-Qiyas A., Lashine Y., Mohammad M., Soliman S.S. (2024). Uniqueness of Candida Auris Cell Wall in Morphogenesis, Virulence, Resistance, and Immune Evasion. Microbiol. Res..

[14] Al-hussaniy H.A., Alburghaif A.H., Naji M.A. (2021). Leptin Hormone and Its Effectiveness in Reproduction, Metabolism, Immunity, Diabetes, Hopes and Ambitions. J. Med. Life.

[15] Dave M. (2025). EBD Spotlight: Antibiotic Resistance in Secondary Endodontic Infections. BDJ Team.

[16] Salako N.O., Rotimi V.O., Adib S.M., Al-Mutawa S. (2004). Pattern of Antibiotic Prescription in the Management of Oral Diseases among Dentists in Kuwait. J. Dent..

[17] Kuriyama T., Absi E.G., Williams D.W., Lewis M.A.O. (2005). An Outcome Audit of the Treatment of Acute Dentoalveolar Infection: Impact of Penicillin Resistance. Br. Dent. J..

[18] Farrier J.N., Kittur M.A., Sugar A.W. (2007). Necrotising Fasciitis of the Submandibular Region; a Complication of Odontogenic Origin. Br. Dent. J..

[19] Ellison S.J. (2009). The Role of Phenoxymethylpenicillin, Amoxicillin, Metronidazole and Clindamycin in the Management of Acute Dentoalveolar Abscesses—A Review. Br. Dent. J..

[20] Abdullah F.M., Hatim Q.Y., Oraibi A.I., Alsafar T.H., Alsandook T.A., Lutfi W., Al-Hussaniy H.A. (2024). Antimicrobial Management of Dental Infections: Updated Review. Medicine.

[21] Darvish S., Budala D.-G., Goriuc A. (2024). Antibacterial Properties of an Experimental Dental Resin Loaded with Gold Nanoshells for Photothermal Therapy Applications. J. Funct. Biomater..

[22] Rubilar-Huenchuman M., Ortega-Villanueva C., González I.A., Palavecino C.E. (2024). The Effect of Photodynamic Therapy on Enterococcus Spp. and Its Application in Dentistry: A Scoping Review. Pharmaceutics.

[23] Lin J., Fang J., Zhou J., Qi M., Shi Y., Li C., Sun X., Dong B., Wang L. (2024). NIR-II Triggered Cu(I) Phosphide for Chemodynamic and Photothermal Periodontitis Treatment: Efficient Reduction of Bacterial Co-Aggregation. Acta Biomater..

[24] Pourhajibagher M., Bahrami R., Bahador A. (2024). Application of Antimicrobial Sonodynamic Therapy as a Potential Treatment Modality in Dentistry: A Literature Review. J. Dent. Sci..

[25] Yu Y.-M., Lu Y.-P., Zhang T., Zheng Y.-F., Liu Y.-S., Xia D.-D. (2024). Biomaterials Science and Surface Engineering Strategies for Dental Peri-Implantitis Management. Mil. Med. Res..

[26] Tian Y., Song Y., Liu J., Lan S., Chen B., Li Y., Han J. (2025). Nanoparticle-Mediated Photothermal and Photodynamic Antibacterial Therapy for the Treatment of Periodontitis. Colloids Surf. Physicochem. Eng. Asp..

[27] He C., Feng P., Hao M., Tang Y., Wu X., Cui W., Ma J., Ke C. (2024). Nanomaterials in Antibacterial Photodynamic Therapy and Antibacterial Sonodynamic Therapy. Adv. Funct. Mater..

[28] Ren H., Cao B., Xu Q., Zhao R., Li H., Wei B. (2025). Role of Microbiota in Pain: From Bench to Bedside. iMetaOmics.

[29] Aherne O., Ortiz R., Fazli M.M., Davies J.R. (2022). Effects of Stabilized Hypochlorous Acid on Oral Biofilm Bacteria. BMC Oral Health.

[30] Chen J., Luo A., Xu M., Zhang Y., Wang Z., Yu S., Zhu L., Wu W., Yang D. (2024). The Application of Phenylboronic Acid Pinacol Ester Functionalized ROS-Responsive Multifunctional Nanoparticles in the Treatment of Periodontitis. J. Nanobiotechnol..

[31] Luo X., Zhang Y., Zeng Y., Yang D., Zhou Z., Zheng Z., Xiao P., Ding X., Li Q., Chen J. (2025). Nanotherapies Based on ROS Regulation in Oral Diseases. Adv. Sci..

[32] Sun X., Mao C., Wang J., Wu S., Qu Y., Xie Y., Sun F., Jiang D., Song Y. (2024). Unveiling the Potential of Sulfur-Containing Gas Signaling Molecules in Acute Lung Injury: A Promising Therapeutic Avenue. Curr. Issues Mol. Biol..

[33] Munteanu C., Turnea M.A., Rotariu M. (2023). Hydrogen Sulfide: An Emerging Regulator of Oxidative Stress and Cellular Homeostasis—A Comprehensive One-Year Review. Antioxidants.

[34] Toledano M., Toledano-Osorio M., Carrasco-Carmona Á., Vallecillo C., Toledano R., Medina-Castillo A.L., Osorio R. (2020). State of the Art on Biomaterials for Soft Tissue Augmentation in the Oral Cavity. Part II: Synthetic Polymers-Based Biomaterials. Polymers.

[35] Gnanasekar S., He X., Nagay B.E., Xu K., Rao X., Duan S., Murugesan S., Barão V.A.R., Kang E.-T., Xu L. (2025). Antibacterial MXenes: An Emerging Non-Antibiotic Paradigm for Surface Engineering of Orthopedic and Dental Implants. Bioact. Mater..

[36] Zhang M., Feng Q., Zhang G., Wang R., Zhang H., Li M., Zhou Y., Jiang C., Li J., Nie Y. (2025). Calcium-Capturing Hydrogel with Self-Reinforced Multi-Dynamic Networks for Effective Periodontal Bone Regeneration in Three-Dimension. Adv. Funct. Mater..

[37] Jiang Z., Javed M.U., Tang X., Qu S., Huang D., Guo B. (2025). Smart Biomaterials in Dentistry: A Review on the Role of Cellulose and Other Biological Macromolecules in Infection Control. Int. J. Biol. Macromol..

[38] Yadav R., Meena A., Patnaik A. (2022). Biomaterials for Dental Composite Applications: A Comprehensive Review of Physical, Chemical, Mechanical, Thermal, Tribological, and Biological Properties. Polym. Adv. Technol..

[39] Parhi S., Pal S., Das S.K., Ghosh P. (2021). Strategies toward Development of Antimicrobial Biomaterials for Dental Healthcare Applications. Biotechnol. Bioeng..

[40] Wang X., Chen Q., Li J., Tian W., Liu Z., Chen T. (2024). Recent Advances of Functional Modules for Tooth Regeneration. J. Mater. Chem. B.

[41] Iwasaki M., Takedachi M., Sawada K., Miki K., Murakami S. (2025). Preservation Therapy for Vertically Fractured Teeth with Periodontal Tissue Regeneration Using FGF-2. Clin. Adv. Periodontics.

[42] Chen Y., Xie J., Gao J., Wang Y., Yue X., Qu J., Ding D., Zhang X., Xin J., Shen J. (2025). Cell Membrane-Coated Nanomicrospheres Mimicking Stem Cell Functions Enhance Angiogenesis for Dental Pulp Regeneration. Mater. Chem. Front..

[43] Umapathy V.R., Natarajan P.M., Swamikannu B. (2025). Regenerative Strategies in Dentistry: Harnessing Stem Cells, Biomaterials and Bioactive Materials for Tissue Repair. Biomolecules.

[44] Soni M., Soni P., Soni P. (2025). Biomimetic Approaches in Prosthodontics: Toward Natural Tooth Restoration and Regeneration. J. Pharm. Bioallied Sci..

[45] Mangal U., Kwon J.-S., Choi S.-H. (2020). Bio-Interactive Zwitterionic Dental Biomaterials for Improving Biofilm Resistance: Characteristics and Applications. Int. J. Mol. Sci..

[46] Shi Y., Sun X., Fang J., Li C., Dong B., Qi M., Wang L. (2025). Multifunctional Nanomaterials for Dental Photo-Theranostics. Chem. Soc. Rev..

[47] Butler J., Handy R.D., Upton M., Besinis A. (2023). Review of Antimicrobial Nanocoatings in Medicine and Dentistry: Mechanisms of Action, Biocompatibility Performance, Safety, and Benefits Compared to Antibiotics. ACS Nano.

[48] Reise M., Kranz S., Heyder M., Beck J., Roth C., Guellmar A., von Eggeling F., Schubert U., Löffler B., Sigusch B. (2023). Salivary Pellicle Formed on Dental Composites Evaluated by Mass Spectrometry—An In Situ Study. Molecules.

[49] Souza J.G.S., Bertolini M., Liu J., Nagay B.E., Martins R., Costa R.C., Brunson J.C., Shibli J., Figueiredo L.C., Dongari-Bagtzoglou A. (2025). Exploring the Impact of Biotic and Abiotic Surfaces on Protein Binding Modulation and Bacteria Attachment: Integrating Biological and Mathematical Approaches. ACS Nano.

[50] Enax J., Ganss B., Amaechi B.T., Schulze zur Wiesche E., Meyer F. (2023). The Composition of the Dental Pellicle: An Updated Literature Review. Front. Oral Health.

[51] Samaranayake L., Tuygunov N., Schwendicke F., Osathanon T., Khurshid Z., Boymuradov S.A., Cahyanto A. (2025). The Transformative Role of Artificial Intelligence in Dentistry: A Comprehensive Overview. Part 1: Fundamentals of AI, and Its Contemporary Applications in Dentistry. Int. Dent. J..

[52] Tuygunov N., Samaranayake L., Khurshid Z., Rewthamrongsris P., Schwendicke F., Osathanon T., Yahya N.A. (2025). The Transformative Role of Artificial Intelligence in Dentistry: A Comprehensive Overview Part 2: The Promise and Perils, and the International Dental Federation Communique. Int. Dent. J..

[53] Sohrabniya F., Hassanzadeh-Samani S., Ourang S.A., Jafari B., Farzinnia G., Gorjinejad F., Ghalyanchi-Langeroudi A., Mohammad-Rahimi H., Tichy A., Motamedian S.R. (2025). Exploring a Decade of Deep Learning in Dentistry: A Comprehensive Mapping Review. Clin. Oral Investig..

[54] Najeeb M., Islam S. (2025). Artificial Intelligence (AI) in Restorative Dentistry: Current Trends and Future Prospects. BMC Oral Health.

[55] Alauddin M.S., Baharuddin A.S., Mohd Ghazali M.I. (2021). The Modern and Digital Transformation of Oral Health Care: A Mini Review. Healthcare.

[56] Sedghi L., DiMassa V., Harrington A., Lynch S.V., Kapila Y.L. (2021). The Oral Microbiome: Role of Key Organisms and Complex Networks in Oral Health and Disease. Periodontology 2000.

[57] Darby I. (2022). Risk Factors for Periodontitis & Peri-implantitis. Periodontology 2000.

[58] Nasiri K., Masoumi S.M., Amini S., Goudarzi M., Tafreshi S.M., Bagheri A., Yasamineh S., Alwan M., Arellano M.T.C., Gholizadeh O. (2023). Recent Advances in Metal Nanoparticles to Treat Periodontitis. J. Nanobiotechnol..

[59] Schierz O., Hirsch C., Krey K.-F., Ganss C., Kämmerer P.W., Schlenz M.A. (2024). DIGITAL DENTISTRY AND ITS IMPACT ON ORAL HEALTH-RELATED QUALITY OF LIFE. J. Evid.-Based Dent. Pract..

[60] Kaan A.M., Kahharova D., Zaura E. (2021). Acquisition and Establishment of the Oral Microbiota. Periodontology 2000.

[61] Chen X., Daliri E.B.-M., Tyagi A., Oh D.-H. (2021). Cariogenic Biofilm: Pathology-Related Phenotypes and Targeted Therapy. Microorganisms.

[62] D’AIuto F., Suvan J., Siripaiboonpong N., Gatzoulis M.A., D’AIuto F. (2025). The Root of the Matter: Linking Oral Health to Chronic Diseases Prevention. Int. J. Cardiol. Congenit. Heart Dis..

[63] Sanz M., del Castillo A.M., Jepsen S., Gonzalez-Juanatey J.R., D’Aiuto F., Bouchard P., Chapple I., Dietrich T., Gotsman I., Graziani F. (2020). Periodontitis and Cardiovascular Diseases: Consensus Report. J. Clin. Periodontol..

[64] Lockhart P.B., Bolger A.F., Papapanou P.N., Osinbowale O., Trevisan M., Levison M.E., Taubert K.A., Newburger J.W., Gornik H.L., Gewitz M.H. (2012). Periodontal Disease and Atherosclerotic Vascular Disease: Does the Evidence Support an Independent Association?. Circulation.

[65] Peikert S.A., Liedtke N.B., Vach K., Streletz E., Rieger S., Palm J., Mittelhamm F., Kirchner S., Hakes P., Gantert L. (2024). Nutrition and Periodontitis: A Cross-Sectional Study from a Practice-Based Research Network. Nutrients.

[66] Romito G.A., Collins J.R., Hassan M.A., Benítez C., Contreras A. (2024). Burden and Impact of Periodontal Diseases on Oral Health-Related Quality of Life and Systemic Diseases and Conditions: Latin America and the Caribbean Consensus 2024. Braz. Oral Res..

[67] Kunath B.J., De Rudder C., Laczny C.C., Letellier E., Wilmes P. (2024). The Oral–Gut Microbiome Axis in Health and Disease. Nat. Rev. Microbiol..

[68] Takahashi N. (2005). Microbial Ecosystem in the Oral Cavity: Metabolic Diversity in an Ecological Niche and Its Relationship with Oral Diseases. Int. Congr. Ser..

[69] Jungbauer G., Stähli A., Zhu X., Alberi L.A., Sculean A., Eick S. (2022). Periodontal Microorganisms and Alzheimer Disease—A Causative Relationship?. Periodontology 2000.

[70] Mosaddad S.A., Mahootchi P., Safari S., Rahimi H., Aghili S.S. (2023). Interactions between Systemic Diseases and Oral Microbiota Shifts in the Aging Community: A Narrative Review. J. Basic. Microbiol..

[71] Jakubovics N.S., Goodman S.D., Mashburn-Warren L., Stafford G.P., Cieplik F. (2021). The Dental Plaque Biofilm Matrix. Periodontology 2000.

[72] Hernández P., Sánchez M.C., Llama-Palacios A., Ciudad M.J., Collado L. (2022). Strategies to Combat Caries by Maintaining the Integrity of Biofilm and Homeostasis during the Rapid Phase of Supragingival Plaque Formation. Antibiotics.

[73] Bertolini M., Costa R.C., Barão V.A.R., Villar C.C., Retamal-Valdes B., Feres M., Silva Souza J.G. (2022). Oral Microorganisms and Biofilms: New Insights to Defeat the Main Etiologic Factor of Oral Diseases. Microorganisms.

[74] Siqueira W.L., Zhang W., Helmerhorst E.J., Gygi S.P., Oppenheim F.G. (2007). Identification of Protein Components in in Vivo Human Acquired Enamel Pellicle Using LC−ESI−MS/MS. J. Proteome Res..

[75] Trautmann S., Künzel N., Fecher-Trost C., Barghash A., Dudek J., Flockerzi V., Helms V., Hannig M. (2022). Is the Proteomic Composition of the Salivary Pellicle Dependent on the Substrate Material?. Proteom.—Clin. Appl..

[76] Chawhuaveang D.D., Yu O.Y., Yin I.X., Lam W.Y.-H., Mei M.L., Chu C.-H. (2021). Acquired Salivary Pellicle and Oral Diseases: A Literature Review. J. Dent. Sci..

[77] Rüdiger S.G., Dahlén G., Carlén A. (2012). Pellicle and Early Dental Plaque in Periodontitis Patients before and after Surgical Pocket Elimination. Acta Odontol. Scand..

[78] Murray P.E., Coffman J.A., Garcia-Godoy F. (2024). Oral Pathogens’ Substantial Burden on Cancer, Cardiovascular Diseases, Alzheimer’s, Diabetes, and Other Systemic Diseases: A Public Health Crisis—A Comprehensive Review. Pathogens.

[79] Huang X., Huang X., Huang Y., Zheng J., Lu Y., Mai Z., Zhao X., Cui L., Huang S. (2023). The Oral Microbiome in Autoimmune Diseases: Friend or Foe?. J. Transl. Med..

[80] Li X., Liu Y., Yang X., Li C., Song Z. (2022). The Oral Microbiota: Community Composition, Influencing Factors, Pathogenesis, and Interventions. Front. Microbiol..

[81] Qi J., Si C., Liu H., Li H., Kong C., Wang Y., Chang B. (2025). Advances of Metal-Based Nanomaterials in the Prevention and Treatment of Oral Infections. Adv. Healthc. Mater..

[82] Rajasekaran J.J., Krishnamurthy H.K., Bosco J., Jayaraman V., Krishna K., Wang T., Bei K. (2024). Oral Microbiome: A Review of Its Impact on Oral and Systemic Health. Microorganisms.

[83] Yamazaki K., Kamada N. (2024). Exploring the Oral-Gut Linkage: Interrelationship between Oral and Systemic Diseases. Mucosal Immunol..

[84] Al-Qadami G., Van Sebille Y., Bowen J., Wardill H. (2022). Oral-Gut Microbiome Axis in the Pathogenesis of Cancer Treatment-Induced Oral Mucositis. Front. Oral Health.

[85] Suárez L.J., Arboleda S., Angelov N., Arce R.M. (2021). Oral Versus Gastrointestinal Mucosal Immune Niches in Homeostasis and Allostasis. Front. Immunol..

[86] Sarfi S., Azaryan E., Naseri M. (2024). Immune System of Dental Pulp in Inflamed and Normal Tissue. DNA Cell Biol..

[87] Pohl S., Akamp T., Smeda M., Uderhardt S., Besold D., Krastl G., Galler K.M., Buchalla W., Widbiller M. (2024). Understanding Dental Pulp Inflammation: From Signaling to Structure. Front. Immunol..

[88] Wen Y.-H., Lin Y.-X., Zhou L., Lin C., Zhang L. (2024). The immune landscape in apical periodontitis: From mechanism to therapy. Int. Endod. J..

[89] Barbero-Navarro I., Irigoyen-Camacho M.E., Zepeda-Zepeda M.A., Ribas-Perez D., Castaño-Seiquer A., Sofian-Pauliuc I. (2024). Understanding the Dynamics of Inflammatory Cytokines in Endodontic Diagnosis: A Systematic Review. Diagnostics.

[90] Li Q. (2025). Diagnostic Utility of Th2 Cytokines (IL-4, IL-5, IL-10, and IL-13) in Pulpal Blood for Assessing Pulpitis Severity. BMC Oral Health.

[91] Zhou W., Huang W., You H., Zhang M., Ma Y., Liu L., Lin M., He S., Huang Y. (2025). EZH2 knockout in mice activates STAT3 signalling via STAT3 methylation and modulates ferroptosis in pulpitis-affected dental pulp vascular endothelial cells: A laboratory investigation. Int. Endod. J..

[92] He X.-T., Li X., Zhang M., Tian B.-M., Sun L.-J., Bi C.-S., Deng D.-K., Zhou H., Qu H.-L., Wu C. (2022). Role of Molybdenum in Material Immunomodulation and Periodontal Wound Healing: Targeting Immunometabolism and Mitochondrial Function for Macrophage Modulation. Biomaterials.

[93] Liu C., Mo L., Niu Y., Li X., Zhou X., Xu X. (2017). The Role of Reactive Oxygen Species and Autophagy in Periodontitis and Their Potential Linkage. Front. Physiol..

[94] Muniz F.W.M.G., Nogueira S.B., Mendes F.L.V., Rösing C.K., Moreira M.M.S.M., de Andrade G.M., Carvalho R.d.S. (2015). The Impact of Antioxidant Agents Complimentary to Periodontal Therapy on Oxidative Stress and Periodontal Outcomes: A Systematic Review. Arch. Oral Biol..

[95] Sui L., Wang J., Xiao Z., Yang Y., Yang Z., Ai K. (2020). ROS-Scavenging Nanomaterials to Treat Periodontitis. Front. Chem..

[96] Mei H., Liu H., Sha C., Lv Q., Song Q., Jiang L., Tian E., Gao Z., Li J., Zhou J. (2024). Multifunctional Metal–Phenolic Composites Promote Efficient Periodontitis Treatment via Antibacterial and Osteogenic Properties. ACS Appl. Mater. Interfaces.

[97] Zhang C., Yan R., Bai M., Sun Y., Han X., Cheng C., Ye L. (2024). Pt-Clusters-Equipped Antioxidase-Like Biocatalysts as Efficient ROS Scavengers for Treating Periodontitis. Small.

[98] Xin X., Liu J., Liu X., Xin Y., Hou Y., Xiang X., Deng Y., Yang B., Yu W. (2024). Melatonin-Derived Carbon Dots with Free Radical Scavenging Property for Effective Periodontitis Treatment via the Nrf2/HO-1 Pathway. ACS Nano.

[99] Li B., Liu F., Ye J., Cai X., Qian R., Zhang K., Zheng Y., Wu S., Han Y. (2022). Regulation of Macrophage Polarization Through Periodic Photo-Thermal Treatment to Facilitate Osteogenesis. Small.

[100] Qiu X., Yu Y., Liu H., Li X., Sun W., Wu W., Liu C., Miao L. (2021). Remodeling the Periodontitis Microenvironment for Osteogenesis by Using a Reactive Oxygen Species-Cleavable Nanoplatform. Acta Biomater..

[101] Tenchov R., Bird R., Curtze A.E., Zhou Q. (2021). Lipid Nanoparticles─From Liposomes to mRNA Vaccine Delivery, a Landscape of Research Diversity and Advancement. ACS Nano.

[102] Mehta M., Bui T.A., Yang X., Aksoy Y., Goldys E.M., Deng W. (2023). Lipid-Based Nanoparticles for Drug/Gene Delivery: An Overview of the Production Techniques and Difficulties Encountered in Their Industrial Development. ACS Mater. Au.

[103] Aschmann D., Knol R.A., Kros A. (2024). Lipid-Based Nanoparticle Functionalization with Coiled-Coil Peptides for In Vitro and In Vivo Drug Delivery. Acc. Chem. Res..

[104] dos Santos D.M., Moon J.-I., Kim D.-S., Bassous N.J., Marangon C.A., Campana-Filho S.P., Correa D.S., Kang M.-H., Kim W.-J., Shin S.R. (2024). Hierarchical Chitin Nanocrystal-Based 3D Printed Dual-Layer Membranes Hydrogels: A Dual Drug Delivery Nano-Platform for Periodontal Tissue Regeneration. ACS Nano.

[105] Chen E., Wang T., Tu Y., Sun Z., Ding Y., Gu Z., Xiao S. (2023). ROS-Scavenging Biomaterials for Periodontitis. J. Mater. Chem. B.

[106] Yu Y., Zhao S., Gu D., Zhu B., Liu H., Wu W., Wu J., Wei H., Miao L. (2022). Cerium Oxide Nanozyme Attenuates Periodontal Bone Destruction by Inhibiting the ROS–NFκB Pathway. Nanoscale.

[107] Wang J., Zhang L., Wang L., Tang J., Wang W., Xu Y., Li Z., Ding Z., Jiang X., Xi K. (2024). Ligand-Selective Targeting of Macrophage Hydrogel Elicits Bone Immune-Stem Cell Endogenous Self-Healing Program to Promote Bone Regeneration. Adv. Healthc. Mater..

[108] Liu X., Hou Y., Yang M., Xin X., Deng Y., Fu R., Xiang X., Cao N., Liu X., Yu W. (2023). N-Acetyl-l-Cysteine-Derived Carbonized Polymer Dots with ROS Scavenging via Keap1-Nrf2 Pathway Regulate Alveolar Bone Homeostasis in Periodontitis. Adv. Healthc. Mater..

[109] Wang H., Wang D., Huangfu H., Lv H., Qin Q., Ren S., Zhang Y., Wang L., Zhou Y. (2022). Branched AuAg Nanoparticles Coated by Metal–Phenolic Networks for Treating Bacteria-Induced Periodontitis via Photothermal Antibacterial and Immunotherapy. Mater. Des..

[110] Lam W., Yao Y., Tang C., Wang Y., Yuan Q., Peng L. (2025). Bifunctional Mesoporous HMUiO-66-NH2 Nanoparticles for Bone Remodeling and ROS Scavenging in Periodontitis Therapy. Biomaterials.

[111] Wu Y., Li X., Sun Y., Tan X., Wang C., Wang Z., Ye L. (2023). Multiscale Design of Stiffening and ROS Scavenging Hydrogels for the Augmentation of Mandibular Bone Regeneration. Bioact. Mater..

[112] Xie Y., Xiao S., Huang L., Guo J., Bai M., Gao Y., Zhou H., Qiu L., Cheng C., Han X. (2023). Cascade and Ultrafast Artificial Antioxidases Alleviate Inflammation and Bone Resorption in Periodontitis. ACS Nano.

[113] Yao H., Turali Emre E.S., Fan Y., Wang J., Liu F., Wei J. (2025). L-Arginine Modified Mesoporous Bioactive Glass with ROS Scavenging and NO Release for Periodontitis Treatment. Bioact. Mater..

[114] Lu Y., Meng Y., Li H., Bai Y., He Y., Heng B.C., Song Y., Han X., Zhang Y., Liang Y. (2025). Self-Bactericidal and Long-Lasting Resin Nanocomposites with Pyrocatalytic Activity Regulated by Oral Temperature Fluctuation. ACS Appl. Mater. Interfaces.

[115] Rong Y., Zhao Z., Lv D., Yin R., Lu L., Xu Z., Ren L., Zhao P., Hu Z., Tao J. (2025). Tailored Metal–Phenolic Network with Hypoglycemic Polyphenol for Promoting Diabetic Wound Healing. ACS Appl. Mater. Interfaces.

[116] Yao K., Zhang Q., Weng L., Li S., Zheng X., Hu L., Luo Y., Huang X., Gong Z., Wang Z. (2024). Cerium-Doped, Alendronate-Loaded, Metal–Organic Framework Nanodrug for Delayed Osteoporosis Progress. ACS Appl. Nano Mater..

[117] Xu Y., Luo Y., Weng Z., Xu H., Zhang W., Li Q., Liu H., Liu L., Wang Y., Liu X. (2023). Microenvironment-Responsive Metal-Phenolic Nanozyme Release Platform with Antibacterial, ROS Scavenging, and Osteogenesis for Periodontitis. ACS Nano.

[118] Zhao Z., Wu C., Huangfu Y., Zhang Y., Zhang J., Huang P., Dong A., Wang Y., Deng J., Wang W. (2024). Bioinspired Glycopeptide Hydrogel Reestablishing Bone Homeostasis through Mediating Osteoclasts and Osteogenesis in Periodontitis Treatment. ACS Nano.

[119] Yu Y., You Z., Li X., Lou F., Xiong D., Ye L., Wang Z. (2024). Injectable Nanocomposite Hydrogels with Strong Antibacterial, Osteoinductive, and ROS-Scavenging Capabilities for Periodontitis Treatment. ACS Appl. Mater. Interfaces.

[120] Li S., Li S., Meng L., Gao R., Liu H., Li M. (2025). Immunopathogenesis and Immunotherapy of Diabetes-Associated Periodontitis. Clin. Oral Investig..

[121] Nakajima M., Kapate N., Clegg J.R., Ikeda-Imafuku M., Park K.S., Kumbhojkar N., Suja V.C., Prakash S., Wang L.L.-W., Tabeta K. (2025). Backpack-Carrying Macrophage Immunotherapy for Periodontitis. J. Control. Release.

[122] Alavi S.E., Ebrahimi Shahmabadi H., Sharma L.A., Sharma A. (2025). Nanoparticle-Based Drug Delivery Systems for Non-Surgical Periodontal Therapy: Innovations and Clinical Applications. 3 Biotech.

[123] Fayazi M., Rostami M., Amiri Moghaddam M., Nasiri K., Tadayonfard A., Roudsari M.B., Ahmad H.M., Parhizgar Z., Majbouri Yazdi A. (2025). A State-of-the-Art Review of the Recent Advances in Drug Delivery Systems for Different Therapeutic Agents in Periodontitis. J. Drug Target..

[124] Swaroop A.E., Mathew S., Harshini P., Nagaraja S. (2025). Local Drug Delivery for Regeneration and Disinfection in Endodontics: A Narrative Review. J. Conserv. Dent. Endod..

[125] Wang P. (2025). Versatile Hybrid Nanoplatforms For Treating Periodontitis And Ros Scavenging. Int. Dent. J..

[126] Zheng Y., Mao L., Wang Q., Hu H., Xarpidin B., Luo Z., Wu Y.-L. (2025). Mitochondria-Targeted ROS Scavenging Natural Enzyme Cascade Nanogels for Periodontitis Treatment via Hypoxia Alleviation and Immunomodulation. Adv. Sci..

[127] Xie C., Zhang Q., Bianco A., Ge S., Ma B. (2025). H2S-Scavenging Hydrogel Alleviating Mitochondria Damage to Control Periodontitis. J. Dent. Res..

